# A multifaceted analysis of HIV-1 protease multidrug resistance phenotypes

**DOI:** 10.1186/1471-2105-12-477

**Published:** 2011-12-15

**Authors:** Kathleen M Doherty, Priyanka Nakka, Bracken M King, Soo-Yon Rhee, Susan P Holmes, Robert W Shafer, Mala L Radhakrishnan

**Affiliations:** 1 Department of Chemistry, Wellesley College, 106 Central Street, Wellesley, MA 02481, USA; 2 Department of Biological Engineering, Massachusetts Institute of Technology, 77 Massachusetts Avenue, Cambridge, MA 02139, USA; 3 Division of Infectious Diseases, Department of Medicine, Stanford University, 300 Pasteur Drive, Stanford, CA, 94305, USA; 4 Department of Statistics, Stanford University, Sequoia Hall, Stanford, CA 94305, USA

## Abstract

**Background:**

Great strides have been made in the effective treatment of HIV-1 with the development of second-generation protease inhibitors (PIs) that are effective against historically multi-PI-resistant HIV-1 variants. Nevertheless, mutation patterns that confer decreasing susceptibility to available PIs continue to arise within the population. Understanding the phenotypic and genotypic patterns responsible for multi-PI resistance is necessary for developing PIs that are active against clinically-relevant PI-resistant HIV-1 variants.

**Results:**

In this work, we use globally optimal integer programming-based clustering techniques to elucidate multi-PI phenotypic resistance patterns using a data set of 398 HIV-1 protease sequences that have each been phenotyped for susceptibility toward the nine clinically-approved HIV-1 PIs. We validate the information content of the clusters by evaluating their ability to predict the level of decreased susceptibility to each of the available PIs using a cross validation procedure. We demonstrate the finding that as a result of phenotypic cross resistance, the considered clinical HIV-1 protease isolates are confined to ~6% or less of the clinically-relevant phenotypic space. Clustering and feature selection methods are used to find representative sequences and mutations for major resistance phenotypes to elucidate their genotypic signatures. We show that phenotypic similarity does not imply genotypic similarity, that different PI-resistance mutation patterns can give rise to HIV-1 isolates with similar phenotypic profiles.

**Conclusion:**

Rather than characterizing HIV-1 susceptibility toward each PI individually, our study offers a unique perspective on the phenomenon of PI class resistance by uncovering major multidrug-resistant phenotypic patterns and their often diverse genotypic determinants, providing a methodology that can be applied to understand clinically-relevant phenotypic patterns to aid in the design of novel inhibitors that target other rapidly evolving molecular targets as well.

## Background

For over fifteen years, drug resistance has been a primary challenge in the effective treatment of HIV, and our understanding of resistance mechanisms has evolved along with the virus itself as new therapies have emerged[1-6]. Thanks to worldwide efforts to tackle HIV drug resistance, many successful treatment regimens have been developed, including combination therapies[7,8] such as the Highly Active Anti-Retroviral Therapy (HAART) regimens[9,10], but treatment options have been uncertain for patients who fail these regimens due to the accumulation of drug-resistant mutations[11]. More recently, in addition to targeting molecules other than HIV-1 reverse transcriptase (RT) and protease, second-generation RT and protease inhibitors (PIs) have been developed such that they remain potent against variants resistant to first-generation inhibitors. Specifically, tipranavir[12] and darunavir[13], the two PIs most recently approved for clinical use, have been shown to be potent against viruses harboring multidrug resistance mutations such as V82A and L90M, in the cases of both tipranavir and darunavir[13-16], and V82T or I84V in the case of darunavir[13,16]. However, even these drugs have been shown to lose potency in the presence of certain mutations or mutation patterns[14,17-20]. In fact, the existence of HIV-1 variants showing resistance to all clinically-approved inhibitors highlights the issue of cross resistance, or the existence of mutation patterns arising from a certain therapeutic regimen that simultaneously cause resistance to other drugs as well. Cross resistance among HIV-1 PIs has been studied[21-26] and reviewed[1,4,27-29] extensively for over a decade, with several key mutation patterns thought to confer cross resistance to the vast majority of PIs. Consequently, one strategy is to take advantage of the lack of cross resistance when a mutation confers resistance to one PI but maintains susceptibility to other PIs. For example, D30N and I50L are associated with resistance specifically to either nelfinavir and atazanavir, respectively, but such mutations do not greatly reduce susceptibility (and I50L actually *increases *susceptibility) to other PIs[30-33]. Sequential or simultaneous administration of regimens that are each potent against variants toward which the other fails may be a potential strategy to prevent drug resistance and treatment failure[34]. In light of the combinatorial number of both potential treatment regimens and potential mutation patterns, it is becoming increasingly important to understand both the major mutation patterns conferring resistance on the genotypic level as well as the major phenotypic patterns of cross resistance - or lack thereof - of these mutation patterns toward the nine clinically-approved PIs.

Computational analyses have played a key role in increasing our understanding of the genotypic and phenotypic patterns of HIV drug resistance and our ability to predict drug response phenotype from genotype[35-37]. The large amount of publicly available data has greatly facilitated these analyses[35,38]. Several computational studies have analyzed new or existing data to identify mutations associated with one or more PI or RT drugs[39-48]. Some studies have presented longitudinal mutagenetic tree or mutation pathway models for the temporal appearances and contingencies of such mutations[49-52]. Others have uncovered pairs or clusters of correlated mutations associated with PI or RT therapy through direct enumeration, statistical or information-theory based methods, clustering, or a combination of techniques[39,43-46,51,53-63]. One particularly successful application of computational analysis is the accurate prediction of drug resistance (phenotype) - often measured as a fold-change in IC_50 _of a drug toward the mutant vs. wild-type - of a target variant given its amino acid sequence (genotype). Many approaches have been used to create prediction models, including regression-based methods[26,64-69], decision trees[70], and other machine learning methods, including artificial neural networks, support vector machines, and others[67,71-74]. Several studies have also comparatively evaluated or combined methods to improve accuracy[67,72,73,75]. Models have also been created for predicting drug resistance phenotype[76] and virological success or failure[77-80] resulting from combination therapies. In addition to these data-driven approaches, structure-based approaches for predicting drug response have also been developed, often in conjunction with the bioinformatics-based approaches[66,81,82]. Taken together, the large collection of available predictive methods still require interpretation and comparison when making patient treatment decisions[83,84], but overall they have been valuable tools both for practical decision-making and for increasing scientific understanding.

The many computational studies of HIV genotype-phenotype data therefore demonstrate the power of uncovering patterns in data, with each study providing a valuable perspective on important features of HIV drug resistance. However, the vast majority of studies have offered a perspective at the genotypic level first - that is, they look for patterns on the genotypic level that correlate with phenotypic responses, usually to one drug or drug regimen at a time, in turn. To our knowledge, a rigorous cluster-based analysis of genotype-phenotype data that first uncovers patterns within the complete *phenotypic *space and *then *determines representative genotypes giving rise to the multidrug response phenotypes has yet to be done. The goal of this study is therefore to provide this unique, simultaneous view into the existing phenotypic patterns amongst all the HIV-1 PIs, as such a perspective can provide novel insights into the major combinations of PIs for which cross resistance can occur.

In this work, we analyze phenotypic drug resistance patterns by considering experimental resistance data of 398 clinical isolates of HIV-1 protease measured against the nine clinically-approved HIV-1 protease inhibitors. To determine phenotypic drug resistance patterns toward all nine drugs, a constrained k-medoids clustering method implemented via integer programming was employed. Clusters were validated by quantifying their ability to predict a sequence's level of resistance toward one drug knowing the sequence's level of resistance toward other drugs. The selection of representative genotypic sequences from each cluster indicated mutations associated with common patterns of phenotypic resistance and can serve as a "panel" of mutants that collectively represent clinically important variants. Furthermore, our direct analysis of phenotypic space allowed us to determine that the virus often utilizes multiple genotypes to achieve similar phenotypic patterns of multidrug resistance. We also show that certain drugs show highly correlated antiviral activities, while other drugs - especially tipranavir - have unique responses. Finally, information theoretic approaches were employed to determine amino acid positions and identities within HIV-1 protease that are most informative for selection into a phenotypic cluster. Taken together, this work provides a simplified framework for understanding major drug resistance patterns toward clinically-approved HIV protease inhibitors and the mutation patterns that best characterize them.

## Methods

### Data set

We analyzed 398 HIV-1 isolates in the HIV Drug Resistance Database[38] (HIVDB) for which cell-based in vitro PI susceptibility testing had been performed by the PhenoSense (Monogram, South San Francisco, CA) assay[85]. Susceptibility was quantified by the Monogram-measured fold-change[85], defined as the ratio of the 50% inhibitory concentration (IC_50_) of the isolate to the IC_50 _of a wild-type control. Only those isolates for which susceptibility had been tested against all nine clinically-approved inhibitors were included. The nine inhibitors considered were amprenavir (APV), atazanavir (ATV), indinavir (IDV), lopinavir (LPV), nelfinavir (NFV), ritonavir (RTV), saquinavir (SQV), tipranavir (TPV), and darunavir (DRV). The data set size was limited by the availability of isolates tested for DRV susceptibility. Many clinical isolates contained mixtures at one or more amino acid positions. Due to the limited data, mixtures were not excluded from the data set. In this work, we will refer to clinical isolates as "sequences," though we recognize that some contain mixtures at certain positions.

To estimate the degree to which mutation frequencies in the genotype/phenotype (n = 398) data set are representative of true population frequencies, the frequencies of non-polymorphic treatment-selected mutations within non-WT sequences were compared between a larger genotype-only data set of 12,290 sequences[38] and the data set used here. Reasonable correlation (Spearman's ρ = 0.88) was found between the data sets (Fig. S1, Additional File 1).

Fold-change values were log-scaled such that for a given drug, a constant factor of fold-change is represented by a constant numerical difference. Because the relationship between fold-change and clinical response is different for each drug, scaled values were standardized so that they represent predicted clinical responses, the phenotype of interest in this work. To do this, the logarithm base used for the log scaling of each drug was set to either the Monogram biological cutoff, the geometric mean of the Monogram lower and upper clinical cutoffs, or the single clinical cutoff provided, depending on which type of cutoff was available for a particular drug (Table 1). Monogram biological cutoffs are defined as the fold-change values below which 99% of the WT sequences reside, and therefore fold-changes above this value likely have decreased susceptibility. Monogram lower and upper clinical cutoffs are fold-change values at which reduced clinical response and unlikely clinical response occur for a given drug, respectively. Ritonavir-boosted cutoff values were used when available. After log-scaling, scaled resistance values of 1 and 0 qualitatively signify decreased susceptibility and susceptibility equal to WT, respectively, for all drugs. To equalize the range of variation in the scaled resistances for each drug and to confine variation to a clinically meaningful range, we capped the maximal and minimal scaled resistances of all drugs to the least extreme value of these among the nine inhibitors -- those of DRV (Table 1). The upper cap of the scaled values (1.83) corresponded to a raw fold-change value for DRV of 500, the upper-limit value used when the fold-change toward DRV was greater than the upper limit of the assay. Sequences with scaled resistances equal to the capped values are therefore considered either highly resistant (upper cap) or potentially hypersusceptible (lower cap). An interpretation of scaled resistance values is in Table 2.

**Table 1:** Scaling and capping of raw fold-change values.

Drug	Base	Max	Min
RTV	2.5	7.30	-1.76

NFV	3.6	4.99	-0.94

ATV	5.2**	3.97	-0.73

APV	6.6*	3.17	-0.85

IDV	10**	2.70	-0.52

LPV	22.3*	2.00	-0.52

SQV	5.3*	4.16	-0.97

TPV	4.0*	4.82	-1.16

DRV	30.0*	1.83*	-0.47*

The bases used for log scaling each drug were informed by Monogram biological and clinical cutoffs as described in the text. Unstarred bases are equal to the available Monogram biological cutoffs; singly-starred bases were calculated as the geometric mean of lower and upper clinical cutoffs. Double-starred bases are equal to the single available Monogram clinical cutoff. For all drugs, scaled values were capped to the least extreme minima and maxima (those of DRV, shown in bold and starred). Maxima in the table were generally used when assay upper limits had been reached, representing that the actual fold resistance was higher than the assay could accurately determine. The maximum measurable fold-change can vary for a given drug between isolates, but due to the cap being well below their ranges of maximum fold-change values, our results are entirely unaffected by variation for most drugs; the upper fold-change limits for DRV and LPV may have at times been slightly lower than the upper cap used here, but as a check for robustness, preliminary results were generated with varying upper caps and were qualitatively similar to those shown here.

**Table 2:** Interpretation of the scaled resistance values used throughout this work.

Scaled resistance	Interpretation	Resistance Score
>1.5	Highly resistant	4

1 to 1.5	Decreased susceptibility	3

0.5 to 1	Slightly decreased susceptibility	2

0 to 0.5	No resistance	1

<= 0	No resistance or hypersusceptibility	0

### Clustering

Sequences were clustered based on their drug-resistance phenotypes, quantified by scaled resistance values. A globally-optimal constrained k-medoids clustering approach was implemented via a linear integer program similar to other variations of integer and mixed-programming-based k-means and k-medoids clustering formulations[86-89]. The k-medoids approach was chosen after exploration of multiple clustering methods (k-means, hierarchical, and a method based on a tight clustering approach[90]), as it was deterministic, provably optimal, and allowed for the easy implementation of hard constraints, which we felt were crucial here for generating clusters that were phenotypically similar across all drugs.

The clustering method was as follows: First, each sequence was assigned a point in a 9-dimensional space whose coordinates are the scaled resistances toward the nine inhibitors. From these points, a distance matrix was generated, in which element d_ij _is the Euclidean 2-norm distance between the i^th ^and j^th ^sequences. The goal was to select k cluster centers (medoids) from within the data set and assign each point in the data set to one of these k medoids such that the sum of the distances from points to their assigned medoids was minimized.

Constraints were placed on this optimization to guarantee phenotypic similarity within a cluster, as the goal of this work is for the clusters to represent major phenotypic patterns. First, a hard constraint was set to bound the distance between any cluster member and its medoid to be less than or equal to a specified value, C. Secondly, a hard constraint was set to cap the maximum infinity norm of the distance between any cluster member and its medoid to a specified value, C_∞_. Such a constraint prohibits grouping together two sequences that are highly similar toward 8 drugs but differ qualitatively in their level of resistance toward only one drug - an undesirable outcome if we wish for our clusters to highlight major cross resistance patterns.

k, the number of clusters, is determined by feasibility; it is the minimum value of clusters for which the constraints are satisfied. In this work we use C = 0.95 and C_∞ _= 0.58; the value of C = 0.95 occurs roughly at the "elbow"[91] or "kink"[92] of a plot of the minimum k needed as a function of tightness (C and C_∞_) (Fig. S2, Additional File 1), suggesting that it allows a reasonable balance between maintaining both a low number of clusters and adequately tight clusters. A C_∞ _of 0.58 guarantees that a given cluster members' scaled resistances toward any given drug cannot vary by more than 2 C_∞ _= 1.16; there will not be a pair of cluster members in which one sequence shows no resistance to a given drug while another shows high levels of resistance (see Table 2). Higher values of C_∞ _would make clusters too diffuse along individual dimensions, preventing their interpretation as clinically-relevant phenotypic patterns. Lower values were found to be too restrictive and generated additional clusters with redundant patterns (data not shown). To check for robustness of clustering as a function of these parameters, C and C_∞ _were each varied in turn up to +/-0.05 units in increments of 0.025. Qualitative phenotypic patterns remained very similar, and pairs of sequences that were clustered together in the original clustering remained together an average of 71% as these parameters were varied.

Figure S3 (Additional File 1) is a plot of the number of clusters (k) vs. data set size, using random subsets of the data. As our data set is currently not large enough to show robust convergence (k increases with increasing data set size), the quantitative results that are affected by data set size are to be considered preliminary; more data could allow for more robust convergence in future studies and would increase confidence in the quantitative conclusions.

The integer programming formulation used is shown in Supplementary Methods (Additional File 1). All integer programs in this work were implemented using the GAMS interface (GAMS Development Corporation, Washington, D.C.) and were solved using CPLEX 11.0.0 (IBM ILOG, Armonk, NY).

### Validation

The clustering was validated by its effectiveness (relative to controls) in predicting the level of drug resistance of a sequence to one drug based on the sequence's levels of drug resistance toward other drugs, using the following n-fold cross-validation procedure[92]:

remove each sequence (in turn) from the data set - label it sequence "A."

cluster the remaining sequences using the above method.

choose one of the nine drugs and eliminate its phenotypic data for sequence "A".

Assign sequence "A" to the cluster to whose centroid it is closest, based on 8-dimensional distance (i.e. removing the eliminated drug's dimension)

Predict the level of drug resistance of sequence A toward the eliminated drug to equal the cluster centroid's scaled resistance value for the eliminated drug. Based on this value, classify sequence A with a resistance score from 0-4 (Table 2).

For each drug, the total RMS error and the percent correctly classified after leaving out each sequence in turn was compared to two controls:

Control 1 ("Random Control"): To predict the resistance of a sequence toward a drug, randomly choose a value from the distribution of scaled resistances in the data set toward the particular drug, and classify it using the corresponding resistance score. This control assumes that the level of resistances between drugs is not correlated.

Control 2 ("Average Control"): To predict the resistance of a sequence toward a given drug, simply use the mean of the levels of sequence "A's" scaled resistances to the other eight drugs, and classify with the corresponding resistance score. This control assumes that resistances toward the nine drugs are highly correlated.

### Genotypic Analyses

In the absence of amino acid mixtures at positions within isolates, the genotypic distance between any two sequences was defined simply as the number of positions at which their amino acid sequence differed. For some analyses, all 99 protease positions were considered. To reduce noise due to polymorphic positions in certain analyses, only 21 positions that have been associated with resistance or drug treatment by previous statistical learning or analysis methods [26,39,48] were considered, unless otherwise noted: 10, 24, 30, 32, 33, 43, 46, 47, 48, 50, 53, 54, 71, 73, 74, 76, 82, 83, 84, 88, and 90. We note that there may be unavoidable arbitrariness in the selection of such a set without considerable initial genotypic-phenotypic analysis (which was exactly what we sought to avoid in this study), and in the course of our research we tried multiple sets, allowing us to check for robustness.

To account for mixtures in isolates, the contribution toward the genotypic difference between two sequences due to a position, d_m_, was defined in the general case as follows:

where "c" is the number of amino acids that the isolates have in common at that position, and max(s) is the number of amino acids in the mixture with the greater number of amino acids at that position. As an example, if one isolate contained a mixture of leucine and methionine at a position and another contained only leucine, then d_m _for this position would be (1-(1/2)) = 1/2.

Intracluster genotypic or phenotypic variability was estimated as the average of all the pairwise genotypic or phenotypic distances. A bootstrapping procedure was used to generate p-values to assess statistical significance of either distance for selected clusters. Random clusters of a size equal to the considered cluster were selected with replacement from the unclustered data, and the distance metrics were calculated. This procedure was repeated 10,000 times to generate distributions for both genotype and phenotype distances, from which p-values were calculated. Bootstrap studentized statistics were obtained by dividing the difference between a value and the bootstrapped distribution mean by the standard deviation of the distribution.

From each cluster, representative sequences were selected. For genotypically diverse clusters, we wished to select multiple representative sequences from each cluster to highlight genotypic diversity. To that end, constrained k-medoids optimizations were run on each cluster using integer programming; the resulting medoids became the representative sequences. For each phenotypic cluster, the minimum value of k was determined such that all sequences within the cluster would be within a genotypic distance of t_i _of at least one medoid. We used a value of t_i _= 9 when possible, as it produced one representative sequence for all but the most diverse clusters (except for other exceptions noted below), allowing for easy interpretability. Additionally, at this k, the sum of the distances between each sequence and its assigned medoid was minimized. Sequences containing mixtures at any of the 21 positions listed above were excluded from being representative, as were sequences with any of the 99 amino acid positions undefined (only 2 within the data set). With this constraint, it becomes possible for phenotypic clusters (other than single-membered ones containing mixtures at relevant positions) not to generate any representative sequences with t_i _= 9. To account for this, t_i _was increased to 10 for clusters 3 and 19 and 10.5 for cluster 10. The integer-programming formulation used here is shown in Supplementary Methods (Additional File 1).

Sets of sequence positions or amino acid residue identities most informative of overall cluster assignment or membership in an individual cluster were identified according to an incremental mutual information (MI)-based method described previously (MIST)[93]. Briefly, the method approximates high-order joint entropies to determine an optimal small subset of features (e.g., residue positions) that collectively have the highest mutual information (MI) with a given output (e.g., phenotypic cluster). These approximated MI values have also been shown to correlate with classification error and with exact MI values in analytically solvable systems. First, the MI between variables of interest was computed, using the frequencies to estimate probabilities. For each MI, the bias in the value was estimated by computing the MI of the pair after randomizing the ordering of the sequence data for each variable 100 times. Variables whose MI with the outputs exceeded their maximum shuffled MI were considered statistically significant and included in subsequent steps; remaining positions were omitted. Sequence positions or binary mutation variables were then selected incrementally to maximize the joint-MI (as estimated by MIST) between the set of all chosen variables and either the cluster assignment or membership in a specific cluster. Mixtures were not included in the distributions. Features were added incrementally until all positions or mutations were included, yielding a full ranking.

### Miscellaneous

Data scaling and other matrix manipulations, including principal component analysis, were done using Matlab 2010a and 2011a (The Mathworks, Natick, MA). Matlab and Microsoft Excel (Microsoft, Inc., Bellevue, WA) using VBA were used for figure generation.

## Results

### Cluster Analysis Reveals Specific Phenotypic Resistance Patterns Among Clinical Isolates

**Figure 1:**
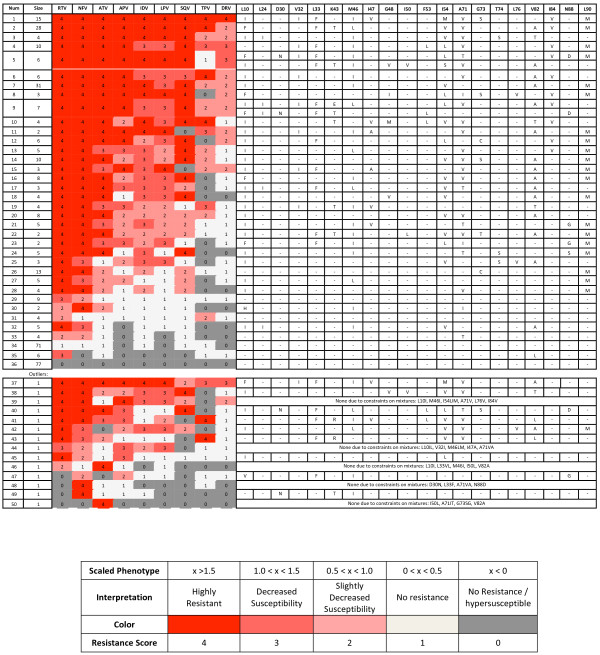
**Optimal phenotypic clustering of clinical data set**. The optimal set of clusters obtained by using constrained k-medoids clustering with integer programming. 36 multi-membered clusters and 14 single-member "clusters", or outliers, were obtained. Each row represents one cluster. The second column indicates the cluster size. The next 9 columns represent the cluster centroids' phenotypic drug resistance scores, colored according to the legend. The columns at right indicate mutations in the sequence selected to represent the cluster at selected positions. Because isolates with mixtures at any of the specified positions were not allowed to represent a cluster, certain single-membered clusters do not have a representative "sequence." The representative sequences chosen for clusters 29, 31, 34, and 36 show no mutations at the positions listed here, but they have substitutions at other positions (Table S1, Additional File 1).

Globally-optimal k-medoids clustering was used to find groups of sequences with similar multidrug phenotypes, using the tightness constraints C and C_∞ _mentioned in the Methods to enforce thresholds of phenotypic similarity. The clustering yielded 36 multi-membered clusters, along with 14 outliers. Figure 1 shows the resulting clusters; each cluster is represented as a row, with each of the colored boxes within the row representing the resistance score (Table 2) toward the corresponding drug of the cluster's centroid (i.e., average phenotype), according to the legend. At right, representative sequences are shown for each cluster, with non-WT amino acid identities shown at selected positions. A listing of mutations at all positions for each representative sequence is provided as Supplementary Information (Table S1, Additional File 1). For two clusters (5 and 9), more than one representative sequence was needed due to the genotypic diversity.

Generally, the largest clusters were those in which (a) there was no resistance (or very mild resistance) to any drug, (b) there was high resistance to all drugs, (c) there was high resistance toward all drugs except DRV, to which there was moderate resistance, (d) there was high resistance toward all drugs except DRV and TPV, (e) there was resistance toward only NFV and RTV, and (f) there was high resistance to APV, ATV, NFV, RTV, and SQV.

The clusters demonstrate that there is often cross resistance of sequences toward many drugs. Generally, sequences are most commonly resistant to RTV and NFV, followed by ATV and SQV, then APV, IND, and LPV, and finally TPV, and DRV. In general, resistance to DRV implies resistance to nearly all other drugs, with a few exceptions: Three clusters showed moderate to high levels of resistance against all drugs except TPV (clusters 5, 8, and 12), and two clusters showed moderate to high levels of resistance against all drugs except SQV (clusters 11 and 15). In both cases, the representative sequences of the clusters each had at least one mutation that has been associated with hypersusceptibility toward the particular drug in a previous study in which mutations were the independent variables and fold-change was the dependent variable[26]. These mutations include L10F, G48V, I50V, I54L, and L76V in the case of the clusters with unique susceptibility to TPV and I47A in the case of the clusters with unique susceptibility to SQV.

One may ask if grouping 398 sequences into 36 phenotypic clusters and 14 outliers shows that HIV is exploring a large or small part of the available phenotypic space. To address this question, we repeatedly generated sets of 398 random points within the same nine-dimensional scaled space of our data set and clustered them using the same constraints applied to the true data set. The average minimum number of clusters needed over 300 trials was 375, with the smallest number of clusters needed being 357. Clearly, the fact that only 50 clusters (including outliers) were needed to partition the actual data within the constraints demonstrates that HIV protease is exploring a very small portion of possible phenotypic space. In fact, due to the constraints used in the clustering, the volume of 9-dimensional phenotypic space occupied by each cluster must be less than the smaller of either the volume of a hypersphere of radius C or a hypercube of length 2C_∞_. Using our constraint values, the smaller of these is the former, with a value of ~2.1 volume units. The volume of clinically-relevant phenotypic space can be calculated from the maximum and minimum scaled values in Table 1 to be 1800 volume units. Therefore, only (2.1*50)/1800 = ~6% of phenotypic space, at best, has been explored by the considered isolates, compared to (2.1*375)/1800 = ~44% for a random data set of equal size.

If a drug is removed from the data set, the minimal number of clusters needed to represent the phenotypic diversity must be less than or equal to the minimal number needed with that drug included. One way to measure the additional phenotypic diversity provided by each drug is to remove each drug in turn and re-cluster using the k-medoids approach under the same distance constraints. Drugs that, upon removal, greatly reduce the number of required clusters have phenotypes that vary somewhat independently from the other drugs. Drugs that, upon removal, do not greatly reduce the number of required clusters have phenotypes that vary predictably with (though not necessarily in a *correlated *manner with) the remaining drugs. When this analysis was carried out, it was found that removal of TPV reduced the number of needed clusters by the most (from 50 to 31), suggesting that TPV's response toward sequences varies somewhat independently from other drugs. In other words, TPV might show varied, graded responses toward certain groups of sequences toward which other drugs show relatively constant responses. Removal of ATV, SQV, or APV also reduced the number of needed clusters by over 10 (from 50 to 37, 38, and 38, respectively). Removal of LPV, DRV, NFV, RTV, or IDV reduced the number of required clusters the least (to 44, 44, 43, 43, and 41, respectively) suggesting that their scaled resistances either vary predictably with those of the other drugs or do not vary appreciably in general.

### Phenotypic clustering allows for potentially improved prediction of unknown drug phenotypes given phenotypic information for other drugs

Our results indicate that a small portion of the full phenotypic space has been explored by the virus, assuming a representative data set; consequently, one may be able to successfully predict resistance to a given inhibitor given resistance data toward other inhibitors, without knowing any genotypic information. To test this hypothesis, we used a cross-validation procedure in which each sequence from the data set was removed in turn and the sequence's resistance toward each drug was estimated based on a clustering assignment using the other eight resistance phenotypes (see Methods). Pairs of sequences that were clustered together in the original clustering remained together an average of 99.3% of the time across all n runs of the validation, not counting runs in which a member of the pair was excluded in turn, demonstrating the stability of the clustering during the cross-validation procedure. The results of the cluster-based prediction are summarized in Table 3.

**Table 3:** Cluster-based prediction of phenotypic resistance relative to controls.

With all data		RTV	NFV	ATV	APV	IDV	LPV	SQV	TPV	DRV
CTL1 (Random)	% correct	35	36	29	21	22	26	29	31	29

CTL1 (Random)	RMSE	1.34	1.13	1.21	1.2	1.05	1.01	1.26	0.98	0.76

CTL2 (Average)	% correct	46	43	62	60	62	56	57	47	34

CTL2 (Average)	RMSE	0.60	0.54	0.36	0.34	0.26	0.28	0.41	0.67	0.67

Cluster-based	% correct	81	75	74	70	63	67	65	50	67

Cluster-based	RMSE	0.35	0.34	0.38	0.33	0.29	0.25	0.50	0.71	0.29

**Without nonresistant clusters**		RTV	NFV	ATV	APV	IDV	LPV	SQV	TPV	DRV

CTL1 (Random)	% correct	78	66	45	27	22	29	34	18	29

CTL1 (Random)	RMSE	0.54	0.68	0.84	0.97	0.86	0.83	1.06	1.00	0.74

CTL2 (Average)	% correct	28	32	49	51	52	43	46	28	11

CTL2 (Average)	RMSE	0.74	0.64	0.45	0.41	0.32	0.34	0.51	0.84	0.84

Cluster-based	% correct	89	82	73	62	55	58	60	34	56

Cluster-based	RMSE	0.26	0.23	0.38	0.40	0.36	0.31	0.62	0.89	0.36

Percent of viruses whose resistance score toward each drug was correctly classified ("% correct"), as well as the RMS error (in scaled resistance units) over all sequences of the phenotypic difference between predicted and actual phenotype ("RMSE") using the two controls described in the text ("CTL1 (Random)" and "CTL2 (Average)" and the cluster-based prediction. The top panel presents results using all 398 sequences, and the bottom panel shows results after removing the two clusters showing little or no phenotypic resistance to any drug.

Two controls were used for comparison and are described in the Methods. Control 1 ("Random"), which randomly reported a value from the distribution of scaled resistances in the data set toward the particular drug, was able to correctly categorize resistance 21%-36% of the time, depending on the drug. The RMSE's of the actual scaled resistance values were often over a whole unit away, meaning that it would often predict no resistance when there was in fact resistance, and vice versa. NFV and RTV were classified correctly most often; the clustering suggests that this may be because they were more likely to exhibit either no resistance or complete resistance, providing a less graded distribution overall from which to sample.

Control 2 ("Average"), which guessed the "unknown" phenotype to be the average of the other 8 known phenotypes for the isolate, performed much better overall than Control 1, categorizing resistance correctly for more than half of the sequences for ATV, APV, IND, LPV, and SQV. Its strong performance is additional evidence for the high level of both correlation between drug responses and cross resistance. Performance was worse for (1) NFV and RTV, which are often inactive to viruses toward which other drugs are effective, as Figure 1 indicates, (2) DRV, which, according to Figure 1, often remains effective toward viruses resistant to other drugs, and (3) TPV, which, as shown above, has less phenotypic similarity to other drugs.

Compared to either control, the cluster-based prediction correctly classified a higher percentage of viruses for every drug, although the improvement over Control 2 was modest in some cases, with the RMSE's being marginally higher in some cases as well, suggesting that when the cluster-based classification was incorrect, it was quite different. The improvement in classification was largest for NFV, RTV, and DRV. Classification rates overall were well over 50% correct with RMS errors being fairly small (generally <= 0.5 units away). The notable exception is TPV, again supporting TPV's uniqueness.

The relatively large number of sequences susceptible to all drugs in our data set might bias the prediction accuracy of certain methods to be higher than what would be expected from a data set that contained a more even distribution of all multidrug phenotypes. To control for this, we redid the above analysis after having left out the sequences corresponding to the two clusters shown in Figure 1 that show no or very little resistance to all nine drugs (clusters 36 and 34, with 77 and 71 members, respectively). Not surprisingly, Control 1 performs much better with RTV and NFV, as now, nearly all sequences in the data set are resistant to either drug. Also unsurprisingly, Control 2 performs worse because the two clusters that were removed contained sequences whose responses to all drugs were highly correlated. The cluster-based classifier still has the highest classification accuracy, but again, the RMSE values were sometimes greater than those for Control 2. Nevertheless, these results show that an understanding of major phenotypic resistance patterns can allow for reasonable prediction of a sequence's resistance toward one drug given resistance information toward other drugs, and the strong performance of the controls under certain circumstances further highlights the underlying structure in the resistance patterns.

### The accumulation of HIV protease mutations results in a "path" in phenotypic space

**Figure 2:**
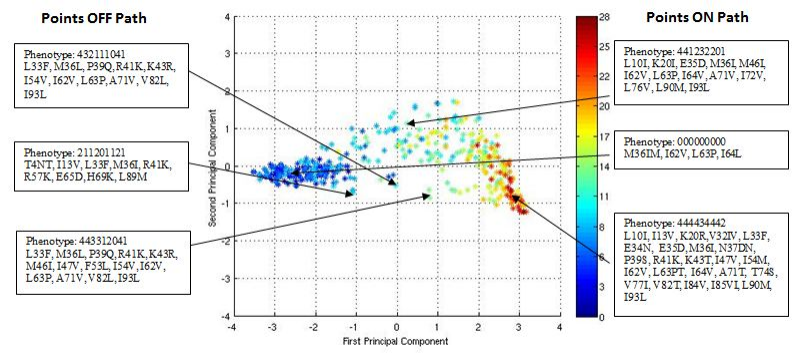
**Projection of the phenotypic data onto its first and second principal components**. Points are colored by the total number of amino acid substitutions relative to the consensus B WT sequence, according to the scale at right; a mixture at a position (including those containing the WT amino acid) is counted as one substitution. The phenotypes and genotypes of selected sequences are indicated. The 9-digit shorthand phenotypic code used to describe the sequences indicates the resistance score (Table 2) to each of the 9 PIs in the order shown in Fig. 1: RTV, NFV, ATV, APV, IDV, LPV, SQV, TPV, DRV. All "outlying" sequences are fully listed in Supplementary Information (Fig. S4, Additional File 1).

Principal component analysis (PCA) was used to project the nine-dimensional, columnwise-centered drug-resistance phenotypes of all sequences onto the two dimensions along which there is most variation. Figure 2 is a plot of the sequences in this two-dimensional space, colored by the total number of amino acid differences from consensus-B wild type protease (considering all 99 amino acid positions). The first two principal components are able to capture approximately 90% of the variation in the data, again suggesting that there are large correlations between drug responses toward the sequences. As indicated in Table 4, the first principal component indicates resistance toward all drugs (i.e., complete cross resistance), with slightly less resistance toward TPV and DRV, relative to their means. The second principal component indicates resistance toward NFV and RTV, less resistance to ATV, SQV, and IDV, and low resistance or even increased susceptibility toward APV, LPV, DRV, and especially TPV, relative to each drug's mean resistance value.

**Table 4:** The nine principal components in scaled phenotypic space.

Principal Component	1	2	3	4	5	6	7	8	9
**Ritonavir**	0.39	0.35	0.60	-0.16	0.30	-0.37	-0.32	-0.05	-0.07

**Nelfinavir**	0.36	0.33	0.22	-0.05	-0.10	0.31	0.75	-0.11	0.17

**Atazanavir**	0.39	0.12	-0.10	0.23	0.01	0.72	-0.49	-0.12	-0.04

**Amprenavir**	0.36	-0.29	-0.17	-0.49	0.29	0.13	0.07	0.63	-0.12

**Indinavir**	0.33	0.04	-0.14	-0.02	-0.62	-0.23	0.02	-0.02	-0.65

**Lopinavir**	0.31	-0.12	-0.18	-0.33	-0.43	-0.19	-0.21	-0.15	0.67

**Saquinavir**	0.38	0.15	-0.48	0.58	0.29	-0.38	0.11	0.14	0.14

**Tipranavir**	0.23	-0.71	0.48	0.43	-0.12	-0.02	0.07	0.07	0.07

**Darunavir**	0.19	-0.37	-0.21	-0.23	0.39	-0.04	0.12	-0.72	-0.21

**Percent Variance (%)**	83.7	6.0	3.1	2.7	1.6	1.0	1.0	0.6	0.3

The first two principal components together capture approximately 90% of the variance in the data and represent development of high resistance toward all drugs and development of high resistance to NFV and RTV with increasing resistance toward ATV, SQV and IDV, respectively.

Interestingly, the points in Figure 2 form a "path" through phenotypic space. Such "horseshoe"-shaped paths are often indicative of a non-linear ordering or underlying gradient in the data[94]. Here, the path clearly tracks the genotypic mutations accrued by the sequences. Sequences with few mutations appear to have resistance toward NFV, RTV, ATV, SQV, and IDV, but little resistance to APV, LPV, DRV, or TPV (i.e., the phenotypic path "veers upward" in the principal component space), while sequences with many mutations are resistant to all drugs (far right in the principal component space). Three sequences along the path are selected in Figure 2 and their corresponding scaled phenotypes and genotypes are listed to the right of the plot. The point selected on the intermediate portion of the path represents a sequence that includes the mutations M46I and L90M, which have been shown to be highly correlated[59] and to be associated with resistance to NFV, IDV, and RTV, and other drugs to a lesser extent[56]. The point selected at the right end of the path represents a sequence that shows at least moderate resistance to all drugs, and includes the mutations V82T, I84V associated with resistance to TPV[18], and L33F, I47V, and I54M, associated with resistance to both TPV[18] and DRV[20], in addition to containing mutations that harbor resistance toward first-generation drugs.

As a whole, Figure 2 supports the historical "path" of drug development, in that it is relatively easy to become resistant to first-generation drugs with relatively few mutations (RTV, NFV, SQV, etc.), but many accumulated mutations appear to be necessary to confer resistance to the newer drugs, such as darunavir[16,19]. Whether or not this pathway is due to history and treatment regimens or whether it is a fundamental consequence of the structural features of the drugs and the viable evolutionary space of HIV-1 protease requires further study.

A handful of sequences lie "off" the pathway. Three such sequences are indicated in Figure 2, and several more are listed in Fig. S4 (Additional file 1). The top and bottom sequences indicated in Figure 2 are both uniquely susceptible to SQV and have the mutation V82L which has been associated with increased SQV susceptibility[26]. The middle sequence shows low levels of resistance across all nine drugs. All three of these sequences fall off the pathway because of their non-negligible levels of resistance toward one or more second-generation drugs while maintaining susceptibility to one or more first-generation drugs. Additional outliers are shown in the Supplementary Information (Additional File 1).

### Phenotypic Similarity Does Not Imply Genotypic Similarity

**Figure 3:**
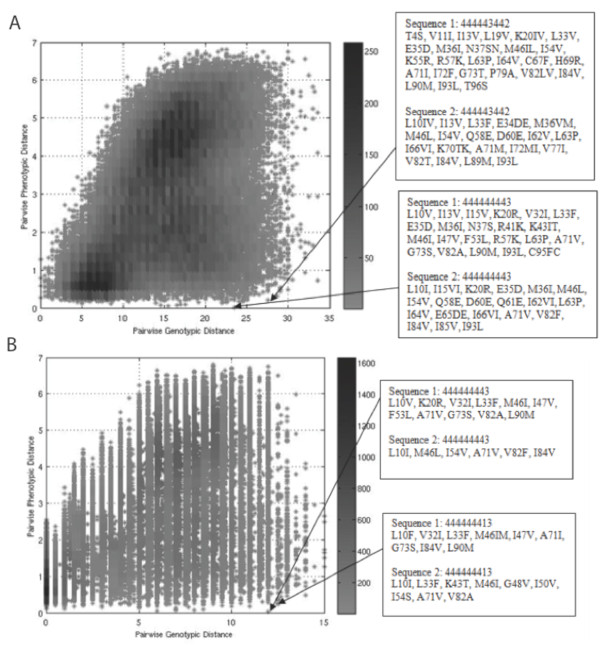
**Pairwise phenotypic distance vs. pairwise genotypic distance for all pairs of sequences**. (a) Scaled phenotypic distance vs. genotypic distance with all positions considered in calculating genotypic distance and (b) with only resistance-associated positions used in calculating genotypic distance. The density of points is colored according to the scale at right. The sequence pairs corresponding to two points are indicated. The 9-digit shorthand used to describe the two pairs of sequences indicates the resistance score (Table 2) to the PIs in the order used in Fig. 1: RTV, NFV, ATV, APV, IDV, LPV, SQV, TPV, DRV.

Figure 3a is a plot of scaled phenotypic distance vs. genotypic distance for all (398*397)/2 = 79003 sequence pairs, using all amino acid positions to compute genotypic distances. Not surprisingly, sequences that are genotypically similar are phenotypically similar; there are no points in the upper-left corner of the plot. However, there are many sequences that are very different genotypically and yet have similar scaled resistance phenotypes (there are many points in the lower-right corner), suggesting that HIV-1 may arrive at the same multidrug resistance phenotype via rather varied genotypes. Figure 3b is again a plot of all pairwise phenotypic distances vs. their genotypic distances, except now, only the resistance-associated positions specified in the Methods have been included in calculating genotypic distance. While the upper left corner of this plot is still sparse, this plot indicates that polymorphic or accessory positions not considered in genotypic distance may still affect resistance profiles in the absence of mutations commonly associated with drug resistance (i.e. there are pairs of sequences with a genotypic distance of zero in Figure 3b but a moderate phenotypic distance). Again, there are still sequences that are genotypically very different yet show similar resistance phenotypes.

Mutations from two sample pairs of sequences from the lower-right quadrant of each figure are shown. In Figure 3b only the mutations contributing to the genotypic distance are shown. As can be seen, very different genotypes can generate similar resistance patterns. For example, the sequences shown in the lower box at the right of Figure 3a show high levels of resistance toward all drugs; each sequence has a subset of documented drug resistance mutations, such as V32I, L33F, M46I, I47V, F53L, G73S, V82A, and L90M in the case of the first sequence and M46L, I54V, V82F, and I84V in the case of the second sequence, but the sequences have few mutations in common (K20R, E35D, M36I, L63P, A71V, and I93L), most of which are considered highly polymorphic accessory mutations[95]. The variety of mutations through which the protease is able to achieve similar multidrug clinical phenotypes demonstrates that phenotypic similarity does not imply genotypic similarity. Recall here that two sequences that are both sufficiently above the clinical fold-change cutoff for resistance for a given drug are both considered phenotypically identical toward that drug, due to the capping of scaled resistance values above a threshold. Therefore, while they are phenotypically similar from a clinical perspective, they may possess quite different (but both large enough to be considered resistant) raw fold-change values toward a given drug.

Another way to understand the genotypic variation for a given phenotypic pattern is to analyze the genotypic diversity within each phenotypic cluster. For each individual phenotypic cluster obtained in the above analysis, we used a k-medoids approach to identify representative genotypes for that cluster. Through constraints, a more genotypically diverse phenotypic cluster would require more sequences to represent it. Figure 1 shows the representative sequences chosen for all phenotypic clusters. As can be seen, two clusters (5 and 9), even though they are of similar sizes to others, require multiple representative genotypic sequences. Multiple representative sequences for a cluster suggest multiple genotypic paths to the phenotype.

To quantify phenotypic and genotypic diversity within clusters, resampling was carried within each cluster as described in the Methods. Table 5 summarizes the results for all clusters with more than 6 members. The p-values for intracluster phenotypic distance ("P Pheno") show significantly low variation, but hard constraints in the clustering enforced phenotypic similarity so this low variation is by design. It is also not surprising that the genotypes of non-resistant clusters are also statistically similar (bootstrap studentized statistics for clusters 34 and 36 are -11.3 and -13.3), as none of these sequences would be expected to bear a resistance-associated mutation, so they should all effectively be "wild-type". However, among multidrug resistant phenotypes, there is either no more or no less genotypic variation between members within a cluster than there is between any two random sequences in the data set (insignificant "P_Geno" values), or there is *more *genotypic variation than would be expected by random sampling in the cases of clusters 5 and 7 (P_Geno < 0.01; bootstrap studentized statistics are 2.26 and 2.16). Furthermore, on average, pairs of sequences from the same cluster generally share less than 50% of their mutations (using resistance-associated positions listed in the Methods); the one exception is the cluster containing sequences resistant to all drugs (cluster 1), whose members share 54% of their mutations on average; indeed the average intra-cluster genotypic distance for this cluster is in some cases less than that for clusters containing fewer mutations on average, suggesting that a higher number of mutations may not mean greater genotypic variation, and also indicating that the most highly resistant sequences might need to have some "key" mutations in common. When removing from the data set one from each pair of 28 sequences from the same patient at two different time points and reclustering, the most highly resistant cluster still had >50% shared mutations on average and a lower intra-cluster genotypic distance than some other resistant clusters, although it now required two representative sequences, suggesting that some - but not all - of this similarity may be due to including data at different time points from the same patient. This idea is further addressed in the Discussion. Nevertheless, while a larger data set would allow for a more rigorous control for the number of mutations within a cluster when computing p-values and for the exclusion of data from the same patients at multiple time points, thus allowing for fairer comparisons, this simple analysis suggests again that in general, phenotypic similarity does not imply genotypic similarity, and certain multidrug phenotypes may be achieved by more varied genotypes than others.

**Table 5:** Statistical analysis of phenotypic and genotypic variability within each cluster containing 6 or more members.

NUM	Phenotype	#Seqs	Intra_Pheno	P_Pheno	Intra_Geno	P_Geno	Avg_Muts	Shared_Muts	**Shan._Ent**.
1	444444444	15	0.69	0	6.15	0.47	9.1	4.95	14.52

2	444444442	28	0.56	0	7.31	0.02	8.3	3.4	18.78

4	444433433	10	0.64	0	7.83	0.05	8.7	3.84	18.33

5	444444413	6	0.63	2E-04	9.63	0.001	9.8	3.9	18.33

6	444433342	6	0.81	7E-04	7.07	0.27	7.7	3.33	13.74

7	444443422	31	0.8	0	7.5	0.008	7	2.43	19

9	444433422	7	0.63	0	8.31	0.04	8.6	3.79	16.91

12	444423402	6	0.65	2E-04	7.82	0.12	7.8	3.04	15.73

14	444232421	10	0.91	1E-04	6.61	0.34	5.9	2.19	14.14

16	444233401	8	0.82	1E-04	6.54	0.38	6.4	2.36	13.84

20	444222221	8	1.01	2E-04	6.18	0.49	5.2	1.41	13.84

26	442121211	13	0.85	0	4.11	0.05	3	0.71	9.88

29	321111111	9	0.8	0	2.38	7E-03	1.3	0.07	5.64

34	111010110	71	0.65	0	0.94	0	0.5	0.03	2.95

35	301000011	6	0.64	2E-04	0.67	3E-03	1	0.67	1.48

36	000000000	77	0.6	0	0.24	0	0.1	0	0.89

"Phenotype" is the nine-digit shorthand describing the binned level of resistance of the cluster centroid toward each of the nine drugs (see Fig. 1 for drug order). "Intra Pheno" is the average intra-cluster phenotypic distance (in scaled resistance unites). "P pheno" are p-values for intra-cluster phenotypic distance. A p-value of 0 indicates that a more extreme distance was not sampled in 10,000 trials. Analogous headings are shown for genotypic distance as well; genotypic distance was defined using the list of non-polymorphic positions in the Methods. "Avg Muts" is the average number of mutations at non-polymorphic positions for sequences within the cluster. "Shared Muts" is the average number of shared mutations between all pairs within a cluster. Shan. Ent. is the computed Shannon Entopy (in bits) for the cluster, adding up the entropies at each non-polymorphic position.

### Feature selection uncovers important positions and mutations for cluster assignment

**Figure 4:**
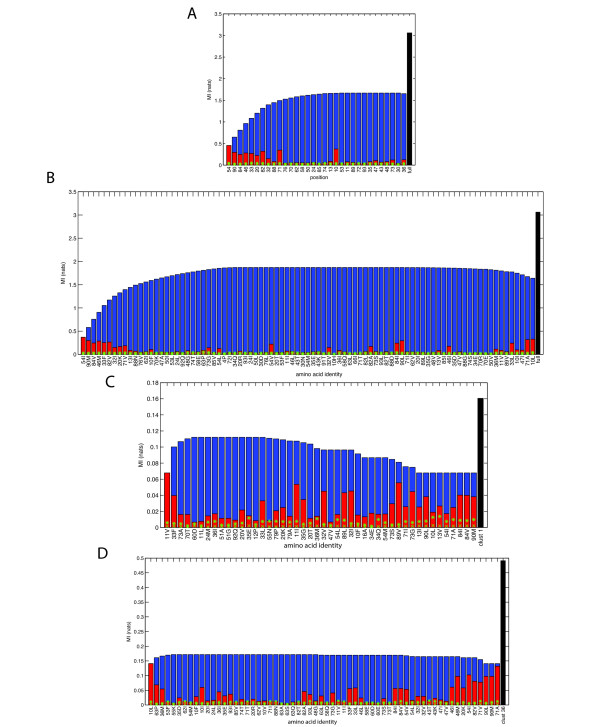
**Stepwise selection of HIV-1 protease positions and mutations that are important for cluster assignment**. (a) Stepwise selection of HIV-1 protease positions (from left to right) such that at each step, the mutual information between amino acids at positions selected so far and the cluster assignment is maximized. Red bars indicate (bias-adjusted) MI between each individual position and cluster assignment. ‘x’s’ are the standard deviation of MI estimation for two independent variables, and asterisks indicate the threshold for statistical significance (p=0.01). Blue bars are the estimated joint MI between the subset chosen and the cluster assignment. The black bar indicates the total information content of the cluster assignments. (b) Stepwise selection of the most informative amino acid identities at specific positions for assignment into phenotypic clusters. (c) and (d): Stepwise selection of particular amino acid identities whose collective presence or absence are maximally informative of membership specifically into cluster 1 (c) and cluster 36 (d).

Finally, we sought to rigorously determine sets of amino acid positions and mutations that were most informative of membership in the phenotypic clusters. Figure 4a shows the results of greedily selecting one position at a time such that at each step (going left to right), the (approximate) mutual information (MI) between the chosen set of features and the cluster assignment is maximized. Only those positions that had significant MI with the output are included. The red bars indicate the MI between an individual position and the cluster assignment, with the yellow star indicating the threshold for statistical significance (p = 0.01). The blue bars indicate the joint MI between the subset selected thus far and the cluster assignment. Note that positions are not strictly selected in decreasing order of individual MI. Because mutations at certain positions may be highly coupled with positions already in the feature set, less individually informative positions may contribute to a more informative set of positions. This technique therefore chooses highly non-redundant features that are still informative of the output. Finally, the black bar shows the total information content of the output, the cluster assignments.

Figure 4a indicates that several positions have significant MI with the final cluster assignment, especially positions 54, 90, 84, 46, 33, 20, 82, 32, 88, and 71. This is consistent with findings that these positions are known to mutate in the presence of drug resistance, either as primary or accessory mutations[4,47,48]. Collectively, these positions are computed to be nearly as informative of ultimate cluster assignments as the entire set of positions considered. The fact that position 54 is chosen as the most informative feature is not surprising, given the large range of drug-resistant mutations commonly found at this position and their varied effects toward certain drugs as either primary or secondary mutations; I54L, I54M, I54V, etc., can have different consequences toward drugs such as TPV, DRV, and APV[4,95] Also interesting is the redundancy of position 10 and, to a lesser extent, position 71; although position 10 has a high mutual information with the cluster output, it does not provide additional information once the identities at the ten positions listed above are known. Position 71 provides some additional information but is also quite redundant. These results are consistent with the amino acids at positions 10 and 71 both being highly correlated with those at other positions such as 54, 90, 82, 84, and others[54,55,59], as it is believed that mutations at these positions can be compensatory in nature[54,55,96]. Finally, one should note that the approximate joint MI calculated between all of the positions and the output is still quite less than the true information content of the output, suggesting that amino acids considered at all positions still may not result in perfect prediction of these output data. This is likely due to the true importance of higher-order information (i.e. patterns of three or more amino acids occurring together) in contributing to ultimate phenotypes - the importance of which has been noted previously[61] - as well as noise in the measurement and clustering of the phenotypic data, thus highlighting the inherent difficulty of accurately predicting phenotype from genotype in these complex systems. The limitations of the second-order approximation also result in the approximated total joint mutual information between the features and the output (blue bars) failing to be monotonically increasing as they would be were an exact calculation feasible, again highlighting the complex relationship between various protease positions and phenotype.

Figure 4b shows the specific amino acid identities calculated to be most informative of ultimate cluster assignment. Here, key resistance mutations are chosen that cause broad resistance to many of the older drugs, such as L90M and I84V. At positions that can bear several identities, such as 54, 46, and 82, the selection of the wild type amino acid suggests the importance of the lack of any mutation at these positions in determining cluster assignment.

Figures 4c and 4d show sample results for mutations that are informative of assignment into specific clusters - cluster 1 (c), the most resistant cluster, and cluster 36 (d), the completely nonresistant cluster. All other results for clusters with 8+ members are shown in Figure S5 (Additional File 1). Figure 4c indicates that the amino acid identities most informative of membership into the "most" resistant cluster include several mutations that have been associated with resistance to DRV[97] including V11I, L33F, V32I, L89V, and G73S, as well as mutations such as I84V and L90M that are associated with broad cross resistance toward other PIs.

Finally, many of the informative residues in the non-resistant cluster (Figure 4d) are actually wild type amino acids. This suggests that the lack of mutations at these positions correlates with low levels of resistance. Additionally, several mutations listed are at accessory positions such as 10, 63 and 36, suggesting that mutations at these positions are reasonable markers for "any" resistance in general. It is important to note that while this method highlights which mutations are most informative of cluster assignment, it does not identify whether it is the presence or the absence of the mutation that is associated with cluster membership.

## Discussion

This study highlighted major patterns of phenotypic resistance across all nine clinically-approved HIV-1 PIs. Cluster analysis yielded several phenotypic patterns, including clusters showing resistance to all drugs, all but one specific drug (such as TPV, SQV, or DRV), a large subset of drugs, a small subset of drugs, and only one drug (such as NFV or ATV). Through choosing representative sequences for each phenotypic pattern, we have corroborated previously reviewed observations[4,27,29] that mutations such as L33F, V82A, I84V, and L90M are associated with broad cross resistance, while others, such as D30N and I50L are associated with resistance to only one drug and still others such as I47A and I54L are linked with hypersusceptibility toward a given drug. While we have uncovered a variety of phenotypic patterns, not every possible resistance pattern was sampled, suggesting that cross resistance and other factors cause highly correlated drug responses, assuming our data set is representative. Indeed, our considered isolates occupy only a small portion (~6%) of the available, clinically-relevant phenotypic space. For example, no cluster shows a moderate or high level of resistance toward DRV without resistance to several other drugs, including APV and LPV. Whether this result is due to patient treatment histories or the intrinsic properties of the drug--protease interactions requires further study. If the latter is at least partly the case, it corroborates the observation that DRV may have a higher genetic barrier to resistance[16,19].

TPV's response toward sequences often shows little relationship to other drugs' responses. The relative lack of cross resistance to TPV may make it particularly useful[14] in conjunction with other inhibitors to "cover" the mutation space of the virus. TPV's differing response profile may follow from its unique structural characteristics. It is the only clinically-approved inhibitor that does not use a water molecule to mediate hydrogen bonds with the flap regions of the protease, suggesting the importance of developing structurally diverse drug molecules toward a target as a strategy to combat resistance[98].

The representative sequences of four clusters (29, 31, 34, and 36) had no mutations at the 21 positions considered in computing genotypic distance for this purpose, and yet their phenotypes were not identical on average. This suggests a potential role for mutations at other positions that may not be associated with primary drug resistance. A rigorous study that analyzes the differences in mutation frequencies in such clusters and considers their impacts on the susceptibilities of individual cluster members, while beyond the scope of the current work, would be interesting potential future work, especially when more data are available.

We demonstrated that phenotypic clustering may allow for prediction of resistance to a particular drug based only on resistance information toward other drugs and no genotypic information. While our goal was not to develop a prediction method that is superior to the available genotypic-based methods specific to each drug, especially as it may be rare to have multidrug phenotypic data available, it is interesting to assess how well our "genotype-blind" method performs when compared to genotype-based methods. Rigorous comparisons to mean standard error values in other studies are difficult due to different scaling and capping procedures used here for phenotypic standardization. Nevertheless, some studies used a Pearson correlation coefficient (R) between predicted and actual log-fold-change as a measure of accuracy. R values for PIs available at the time of selected studies ranged from 0.85-0.97[69], 0.65-0.93 (across multiple methods)[67], and 0.78-0.89[64]. From the cross-validation procedure used to generate Table 3, our "genotype-blind" method gave R-values ranging from 0.84-0.94 using all 398 data set members, with the exception of TPV, although these numbers may be artificially high due to our capping of extreme values. Predictions of resistance to TPV had an R value of only 0.45, consistent with the observed difficulty in predicting TPV resistance based on the phenotypes shown toward other drugs. Finally, our reported classification accuracies are lower than those reported for genotype-based predictions, but this is partly because we use five categories as opposed to the binary or 3-way classifications commonly used. If we adopt a naive binary classification scheme (scaled resistance < 1.0 is not resistant; scaled resistance >= 1.0 is resistant), our cluster-based classification accuracies using the n-fold cross validation procedure for the entire data set range from 85%-95% excluding TPV(79%), compared with 85%-95% for binary classification schemes reported in the literature [65,72,74] (TPV and DRV were not part of these studies). It is interesting to note that while not the major goal of our paper, we have shown that with the exception of TPV, it may be possible to approach comparable drug resistance prediction accuracy without any genotypic information; this level of accuracy demonstrates the restricted phenotypic space occupied by the virus.

Our analysis was limited by the number of accessible isolates that have each undergone phenotypic resistance testing against all nine inhibitors. A large priority for future work is acquiring enough data such that the number of clusters is robust to the data set size such that one could be confident that all or nearly all phenotypic patterns have been sampled. One strategy is to pool isolates phenotyped by different assays to bolster the amount of data; indeed, a preliminary clustering was carried out in which the data analyzed here were combined with 196 isolates phenotyped using the Antivirogram (Virco, Mechelin, Belgium)[99] assay, but differences between the assays may have subtle but important effects on the interpretation of scaled resistance values, even when using cutoffs specific to each assay, creating potential artifacts in the clustering (we obtained 67 clusters with the combined data set, a larger number than expected given the pattern shown in Fig. S3). More data would allow for larger cluster sizes in general, and therefore a higher confidence in associating certain genotypic features with cluster assignments; one could also look for differences in phenotypes between virus subtypes if such data were concurrently available. Additionally, more data may allow for cluster sizes to accurately represent the relative frequencies of phenotypes within the population and would allow us to exclude isolates containing mixtures at key positions; such an exclusion would have been too restrictive with the amount of data currently accessible.

Finally, larger clusters would also allow us to account for and potentially exclude sequences that may be from the same patient at different times, allowing for more robust conclusions to be made about the genotypic variability within a cluster. Preliminary analyses were conducted in which one sequence each from 28 sequences pairs from the same patient in our data set was arbitrarily excluded (even if the pair differed significantly in genotype), yielding a 370-member set. Qualitative results of genotypic variability remained similar, in that several resistant clusters showed as much or more genotypic diversity than randomly chosen data set members, although again, the most resistant cluster showed a higher percentage of shared mutations between cluster members on average even though it now required two representative sequences. 48 clusters were needed to cluster the "unique-patient" data set as opposed to 50 for the original data set, suggesting that data from the same patient taken at different time points can provide additional phenotypic diversity. 98% of sequence pairs grouped together in the smaller data set were grouped together in the original data set, showing that the overall clustering remained very similar.

Since the time the manuscript had been originally drafted, we obtained approximately 50 more isolates, and we have carried out very preliminary analyses of a larger (n = 453) data set including these new sequences. 52 clusters were needed to group the data using the same constraints with the original data set, and the phenotypic patterns of most clusters were identical or highly similar; 86% of sequences pairs that had been grouped together originally remained together in the clustering of the larger data set. We also used our original (n = 398) clusters to predict resistance to each drug for each of the new isolates, using the other drugs' resistance values to select the closest centroid (i.e., the same procedure used in the n-fold cross validation). Scaled resistance scores (0-4) were predicted correctly from 66%-82% of the time, depending on the drug; interestingly, predictions for TPV (67%) and DRV (82%) were better than seen in the n-fold cross validation, while those for NFV (66%) and RTV (76%) were worse. Prediction accuracy may be affected by the points in time at which the data were obtained, as resistance patterns may change over time.

Treatment histories were not entirely available for the current data set; acquiring such information and analyzing future data in their context can provide additional insights. For example, one could determine the extent to which treatment histories affect the "path" seen in Figure 2 and the dependence on individual multidrug resistance phenotypes on past treatment; such analyses could highlight the extent to which treatment histories affect the genotypic variation within a phenotypic cluster.

While the methodology and analyses were applied here to the HIV-1 protease system, the framework is generally applicable to any system for which there are phenotypic data across multiple drugs. In addition to continuing to analyze HIV-1 protease as the available data grow, another natural next step is to apply these methods to the HIV-1 nucleoside or non-nucleoside reverse transcriptase inhibitor systems and to compare the patterns of cross resistance within those systems with the ones obtained in the present study. By rigorously studying phenotypic resistance patterns of multiple systems, one may begin to address more general ideas, including whether cross resistance has equally affected all target systems and whether potential genotypic diversity within phenotypic clusters is a general feature of target systems.

## Conclusions

To our knowledge, this study provided the first cluster-based analysis of the clinically-explored multidrug *phenotypic *space of HIV-1 protease, uncovering major multidrug patterns of resistance, cross resistance, and potential hypersusceptibility. We showed that while genotypic similarity implies clinical phenotypic similarity, the converse is not necessarily the case. We also provided genotypic determinants of phenotypic patterns. Rather than consider each drug in turn, as others have done, we have accounted for their relationships and collapsed the vast nine dimensional space into a smaller one through clustering, allowing us to consider genotypic features that are associated with a simultaneous nine-drug response. We have therefore provided a new perspective on existing drug resistance patterns and their associated genotypic features. Such a framework will be useful as new therapies emerge and will require evaluation in the context of existing drug resistance.

## Authors' contributions

RWS and MLR conceptualized the research. KMD, PN, BMK, SYR, and MLR implemented methods and performed analyses. SYR and RWS acquired data. SPH provided critical support for certain analyses. RWS, BMK, PN, and MLR drafted the manuscript. All authors have read and approved the final manuscript.

## Supplementary Material

Additional file 1 **Contains the formal integer-programming formulations used within the work, five supplementary figures (Figures S1-S5) and one supplementary table (Table S1)**. This file also contains a link to a website containing the n = 398 data set used in this work. (http://www.wellesley.edu/Chemistry/Radhakrishnan/projects.html). Click here for file

## References

[B1] Shafer RW Genotypic testing for human immunodeficiency virus type 1 drug resistance Clinical Microbiology Reviews 2002 15 247 277 10.1128/CMR.15.2.247-277.2002 11932232 PMC118066

[B2] Burlet S Pietrancosta N Laras Y Garino C Quelever G Kraus JL Prospects for the resistance to HIV protease inhibitors: Current drug design approaches and perspectives Current Pharmaceutical Design 2005 11 3077 3090 10.2174/1381612054864939 16178746

[B3] Yin PD Das D Mitsuya H Overcoming HIV drug resistance through rational drug design based on molecular, biochemical, and structural profiles of HIV resistance Cellular and Molecular Life Sciences 2006 63 1706 1724 10.1007/s00018-006-6009-7 16715409 PMC11136232

[B4] Kim R Baxter JD Protease inhibitor resistance update: Where are we now? AIDS Patient Care and Stds 2008 22 267 277 10.1089/apc.2007.0099 18422460

[B5] Shafer RW Schapiro JM HIV-1 drug resistance mutations: an updated framework for the second decade of HAART AIDS Reviews 2008 10 67 84 18615118 PMC2547476

[B6] Ali A Bandaranayake RM Cai YF King NM Kolli M Mittal S Murzycki JF Nalam MNL Nalivaika EA Ozen A Prabu-Jeyabalan MM Thayer K Schiffer CA Molecular Basis for Drug Resistance in HIV-1 Protease Viruses-Basel 2010 2 2509 2535 10.3390/v2112509 PMC3185577 21994628

[B7] Palella FJ Delaney KM Moorman AC Loveless MO Fuhrer J Satten GA Aschman DJ Holmberg SD Declining morbidity and mortality among patients with advanced human immunodeficiency virus infection New England Journal of Medicine 1998 338 853 860 10.1056/NEJM199803263381301 9516219

[B8] Staszewski S Morales-Ramirez J Tashima KT Rachlis A Skiest D Stanford J Stryker R Johnson P Labriola DF Farina D Manion DJ Ruiz NM Efavirenz plus zidovudine and lamivudine, efavirenz plus indinavir, and indinavir plus zidovudine and lamivudine in the treatment of HIV-1 infection in adults New England Journal of Medicine 1999 341 1865 1873 10.1056/NEJM199912163412501 10601505

[B9] Bonfanti P Capetti A Rizzardini G HIV disease treatment in the era of HAART Biomedicine & Pharmacotherapy 1999 53 93 105 10.1016/S0753-3322(99)80066-3 10337464

[B10] Murphy EL Collier AC Kalish LA Assmann SF Para MF Flanigan TP Kumar PN Mintz L Wallach FR Nemo GJ Highly active antiretroviral therapy decreases mortality and morbidity in patients with advanced HIV disease Annals of Internal Medicine 2001 135 17 26 11434728 10.7326/0003-4819-135-1-200107030-00005

[B11] Deeks SG Treatment of anti retroviral-drug-resistant HIV-1 infection Lancet 2003 362 2002 2011 10.1016/S0140-6736(03)15022-2 14683662

[B12] Turner SR Strohbach JW Tommasi RA Aristoff PA Johnson PD Skulnick HI Dolak LA Seest EP Tomich PK Bohanan MJ Horng MM Lynn JC Chong KT Hinshaw RR Watenpaugh KD Janakiraman MN Thaisrivongs S Tipranavir (PNU-140690): A potent, orally bioavailable nonpeptidic HIV protease inhibitor of the 5,6-dihydro-4-hydroxy-2-pyrone sulfonamide class Journal of Medicinal Chemistry 1998 41 3467 3476 10.1021/jm9802158 9719600

[B13] Koh Y Nakata H Maeda K Ogata H Bilcer G Devasamudram T Kincaid JF Boross P Wang YF Ties YF Volarath P Gaddis L Harrison RW Weber IT Ghosh AK Mitsuya H Novel bis-tetrahydrofuranylurethane-containin nonpeptidic protease inhibitor (PI) UIC-94017 (TMC114) with potent activity against multi-PI-resistant human immunodeficiency virus in vitro Antimicrob Agents Chemother 2003 47 3123 3129 10.1128/AAC.47.10.3123-3129.2003 14506019 PMC201142

[B14] Larder BA Hertogs K Bloor S van den Eynde C DeCian W Wang YY Freimuth WW Tarpley G Tipranavir inhibits broadly protease inhibitor-resistant HIV-1 clinical samples AIDS 2000 14 1943 1948 10.1097/00002030-200009080-00009 10997398

[B15] Rusconi S Catamancio SLA Citterio P Kurtagic S Violin M Balotta C Moroni M Galli M D'Arminio-Monforte A Susceptibility to PNU-140690 (Tipranavir) of human immunodeficiency virus type 1 isolates derived from patients with multidrug resistance to other protease inhibitors Antimicrob Agents Chemother 2000 44 1328 1332 10.1128/AAC.44.5.1328-1332.2000 10770770 PMC89863

[B16] Lefebvre E Schiffer CA Resilience to resistance of HIV-1 protease inhibitors: Profile of darunavir AIDS Reviews 2008 10 131 142 18820715 PMC2699666

[B17] Doyon L Tremblay S Bourgon L Wardrop E Cordingley MG Selection and characterization of HIV-1 showing reduced susceptibility to the non-peptidic protease inhibitor tipranavir Antiviral Research 2005 68 27 35 10.1016/j.antiviral.2005.07.003 16122817

[B18] Baxter JD Schapiro JM Boucher CAB Kohlbrenner VM Hall DB Scherer JR Mayers DL Genotypic changes in human immunodeficiency virus type 1 protease associated with reduced susceptibility and virologic response to the protease inhibitor tipranavir J Virol 2006 80 10794 10801 10.1128/JVI.00712-06 16928764 PMC1641746

[B19] De Meyer S Vangeneugden T Lefebvre E Azijn H De Baere I Van Baelen B de Bethune MP Phenotypic and genotypic determinants of resistance to TMC114: Pooled analysis of POWER 1, 2 and 3 Antiviral Therapy 2006 11 S83 S83

[B20] Mitsuya Y Liu TF Rhee SY Fessel WJ Shafer RW Prevalence of darunavir resistance-associated mutations: Patterns of occurrence and association with past treatment Journal of Infectious Diseases 2007 196 1177 1179 10.1086/521624 17955436 PMC2597352

[B21] Tisdale M Myers RE Maschera B Parry NR Oliver NM Blair ED Cross-resistance analysis of human-immunodeficiency-virus type-1 variants individually selected for resistance to 5 different protease inhibitors Antimicrob Agents Chemother 1995 39 1704 1710 7486905 10.1128/aac.39.8.1704 PMC162812

[B22] Hertogs K Bloor S Kemp SD Van den Eynde C Alcorn TM Pauwels R Van Houtte M Staszewski S Miller V Larder BA Phenotypic and genotypic analysis of clinical HIV-1 isolates reveals extensive protease inhibitor cross-resistance: a survey of over 6000 samples AIDS 2000 14 1203 1210 10.1097/00002030-200006160-00018 10894285

[B23] Parkin NT Chappey C Maranta M Whitehurst N Petropoulos CJ Genotypic and Phenotypic Analysis of a Large Database of Patient Samples Reveals Distinct Patterns of Protease Inhibitor Cross-Resistance[abstract] Antiviral Therapy 2001 6 Suppl 1 49

[B24] Harrigan PR Larder BA Extent of cross-resistance between agents used to treat human immunodeficiency virus type 1 infection in clinically derived isolates Antimicrob Agents Chemother 2002 46 909 912 10.1128/AAC.46.3.909-912.2002 11850286 PMC127474

[B25] Watkins T Resch W Irlbeck D Swanstrom R Selection of high-level resistance to human immunodeficiency virus type 1 protease inhibitors Antimicrob Agents Chemother 2003 47 759 769 10.1128/AAC.47.2.759-769.2003 12543689 PMC151730

[B26] Rhee SY Taylor J Fessel WJ Kaufman D Towner W Troia P Ruane P Hellinger J Shirvani V Zolopa A Shafer RW HIV-1 Protease Mutations and Protease Inhibitor Cross-Resistance Antimicrob Agents Chemother 2010 54 4253 4261 10.1128/AAC.00574-10 20660676 PMC2944562

[B27] Race E Cross-resistance within the protease inhibitor class Antiviral Therapy 2001 6 29 36 11678476

[B28] Yusa K Harada S Acquisition of multi-PI (protease inhibitor) resistance in HIV-1 in vivo and in vitro Current Pharmaceutical Design 2004 10 4055 4064 10.2174/1381612043382477 15579087

[B29] Kozal M Cross-resistance patterns among HIV protease inhibitors AIDS Patient Care and Stds 2004 18 199 208 10.1089/108729104323038874 15142350

[B30] Patick AK Duran M Cao Y Shugarts D Keller MR Mazabel E Knowles M Chapman S Kuritzkes DR Markowitz M Genotypic and phenotypic characterization of human immunodeficiency virus type 1 variants isolated from patients treated with the protease inhibitor nelfinavir Antimicrob Agents Chemother 1998 42 2637 2644 9756769 10.1128/aac.42.10.2637 PMC105911

[B31] Colonno R Rose R McLaren C Thiry A Parkin N Friborg J Identification of I50L as the signature atazanavir (ATV)-resistance mutation in treatment-naive HIV-1-infected patients receiving ATV-containing regimens Journal of Infectious Diseases 2004 189 1802 1810 10.1086/386291 15122516

[B32] Yanchunas J Langley DR Tao L Rose RE Friborg J Colonno RJ Doyle ML Molecular basis for increased susceptibility of isolates with atazanavir resistance-conferring substitution I50L to other protease inhibitors Antimicrob Agents Chemother 2005 49 3825 3832 10.1128/AAC.49.9.3825-3832.2005 16127059 PMC1195399

[B33] Weinheimer S Discotto L Friborg J Yang H Colonno R Atazanavir signature I50L resistance substitution accounts for unique phenotype of increased susceptibility to other protease inhibitors in a variety of human immunodeficiency virus type 1 genetic backbones Antimicrob Agents Chemother 2005 49 3816 3824 10.1128/AAC.49.9.3816-3824.2005 16127058 PMC1195397

[B34] Kemper CA Witt MD Keiser PH Dube MP Forthal DN Leibowitz M Smith DS Rigby A Hellmann NS Lie YS Leedom J Richman D McCutchan JA Haubrich R Sequencing of protease inhibitor therapy: insights from an analysis of HIV phenotypic resistance in patients failing protease inhibitors AIDS 2001 15 609 615 10.1097/00002030-200103300-00010 11316998

[B35] Beerenwinkel N Sing T Lengauer T Rahnenfuhrer J Roomp K Savenkov I Fischer R Hoffmann D Selbig J Korn K Walter H Berg T Braun P Fatkenheuer G Oette M Rockstroh J Kupfer B Kaiser R Daumer M Computational methods for the design of effective therapies against drug resistant HIV strains Bioinformatics 2005 21 3943 3950 10.1093/bioinformatics/bti654 16144807

[B36] Lengauer T Sing T Innovation - Bioinformatics-assisted anti-HIV therapy Nature Reviews Microbiology 2006 4 790 U799 10.1038/nrmicro1477 16980939

[B37] Cordes F Kaiser R Selbig J Bioinformatics approach to predicting HIV drug resistance Expert Review of Molecular Diagnostics 2006 6 207 215 10.1586/14737159.6.2.207 16512780

[B38] Rhee SY Gonzales MJ Kantor R Betts BJ Ravela J Shafer RW Human immunodeficiency virus reverse transcriptase and protease sequence database Nucleic Acids Research 2003 31 298 303 10.1093/nar/gkg100 12520007 PMC165547

[B39] Wu TD Schiffer CA Gonzales MJ Taylor J Kantor R Chou SW Israelski D Zolopa AR Fessel WJ Shafer RW Mutation patterns and structural correlates in human immunodeficiency virus type 1 protease following different protease inhibitor treatments J Virol 2003 77 4836 4847 10.1128/JVI.77.8.4836-4847.2003 12663790 PMC152121

[B40] Chen LM Perlina A Lee CJ Positive selection detection in 40,000 human immunodeficiency virus (HIV) type 1 sequences automatically identifies drug resistance and positive fitness mutations in HIV protease and reverse transcriptase J Virol 2004 78 3722 3732 10.1128/JVI.78.7.3722-3732.2004 15016892 PMC371046

[B41] Kantor R Katzenstein DA Efron B Carvalho AP Wynhoven B Cane P Clarke J Sirivichayakul S Soares MA Snoeck J Pillay C Rudich H Rodrigues R Holguin A Ariyoshi K Bouzas MB Cahn P Sugiura W Soriano V Brigido LF Grossman Z Morris L Vandamme AM Tanuri A Phanuphak P Weber JN Pillay D Harrigan PR Camacho R Schapiro JM Shafer RW Impact of HIV-1 subtype and antiretroviral therapy on protease and reverse transcriptase genotype: Results of a global collaboration PLoS Medicine 2005 2 325 337 10.1371/journal.pmed.0020112 PMC1087220 15839752

[B42] Rhee SY Fessel WJ Zolopa AR Hurley L Liu T Taylor J Nguyen DP Slome S Klein D Horberg M Flamm J Follansbee S Schapiro JM Shafer RW HIV-1 protease and reverse-transcriptase mutations: Correlations with antiretroviral therapy in subtype B isolates and implications for drug-resistance surveillance Journal of Infectious Diseases 2005 192 456 465 10.1086/431601 15995959 PMC2597526

[B43] Sing T Svicher V Beerenwinkel N Ceecherini-Silberstein F Daumer M Kaiser R Walter H Korn K Hoffmann D Oette M Rockstroh JK Fatkenheuer G Perno CF Lengauer T Jorge A, Torgo L, Brazdil P, Camacho R, Gama J Characterization of novel HIV drug resistance mutations using clustering, multidimensional scaling and SVM-based feature ranking Knowledge Discovery in Databases: Pkdd 2005 2005 3721 285 296 10.1007/11564126_30

[B44] Svicher V Ceccherini-Silberstein F Erba F Santoro M Gori C Bellocchi MC Giannella S Trotta MP Monforte AD Antinori A Pern CF Novel human immunodeficiency virus type 1 protease mutations potentially involved in resistance to protease inhibitors Antimicrob Agents Chemother 2005 49 2015 2025 10.1128/AAC.49.5.2015-2025.2005 15855527 PMC1087636

[B45] Kagan RM Cheung PK Huard TK Lewinski MA Increasing prevalence of HIV-1 protease inhibitor-associated mutations correlates with long-term non-suppressive protease inhibitor treatment Antiviral Research 2006 71 42 52 10.1016/j.antiviral.2006.02.008 16600392

[B46] Garriga C Perez-Elias MJ Delgado R Ruiz L Najera R Pumarola T Alonso-Socas MD Garcia-Bujalance S Menendez-Arias L Mutational patterns and correlated amino acid substitutions in the HIV-1 protease after virological failure to nelfinavir- and lopinavir/ritonavir-based treatments Journal of Medical Virology 2007 79 1617 1628 10.1002/jmv.20986 17854027

[B47] Shafer RW Rhee SY Pillay D Miller V Sandstrom P Schapiro JM Kuritzkes DR Bennett D HIV-1 protease and reverse transcriptase mutations for drug resistance surveillance AIDS 2007 21 215 223 10.1097/QAD.0b013e328011e691 17197813 PMC2573394

[B48] Bennett DE Camacho RJ Otelea D Kuritzkes DR Fleury H Kiuchi M Heneine W Kantor R Jordan MR Schapiro JM Vandamme AM Sandstrom P Boucher CAB van de Vijver D Rhee SY Liu TF Pillay D Shafer RW Drug Resistance Mutations for Surveillance of Transmitted HIV-1 Drug-Resistance: 2009 Update PLoS One 2009 4 e4724 10.1371/journal.pone.0004724 19266092 PMC2648874

[B49] Beerenwinkel N Daumer M Sing T Rahnenfuhrer J Lengauer T Selbig J Hoffmann D Kaiser R Estimating HIV evolutionary pathways and the genetic barrier to drug resistance Journal of Infectious Diseases 2005 191 1953 1960 10.1086/430005 15871130

[B50] Beerenwinkel N Drton M A mutagenetic tree hidden Markov model for longitudinal clonal HIV sequence data Biostatistics 2007 8 53 71 16569743 10.1093/biostatistics/kxj033

[B51] Deforche K Camacho R Grossman Z Silander T Soares MA Moreau Y Shafer RW Van Laethem K Carvalho AP Wynhoven B Cane P Snoeck J Clarke J Sirivichayakul S Ariyoshi K Holguin A Rudich H Rodrigues R Bouzas MB Cahn P Brigido LF Soriano V Sugiura W Phanuphak P Morris L Weber J Pillay D Tanuri A Harrigan PR Shapiro JM Katzenstein DA Kantor R Vandamme AM Bayesian network analysis of resistance pathways against HIV-1 protease inhibitors Infection Genetics and Evolution 2007 7 382 390 10.1016/j.meegid.2006.09.004 17127103

[B52] Beerenwinkel N Sullivant S Markov models for accumulating mutations Biometrika 2009 96 645 661 10.1093/biomet/asp023

[B53] Gonzales MJ Belitskaya I Dupnik KM Rhee SY Shafer RW Protease and reverse transcriptase mutation patterns in HIV type 1 isolates from heavily treated persons: Comparison of isolates from Northern California with isolates from other regions Aids Res Hum Retrovir 2003 19 909 915 10.1089/088922203322493085 14601580 PMC2547469

[B54] Hoffman NG Schiffer CA Swanstrom R Covariation of amino acid positions in HIV-1 protease Virology 2003 314 536 548 10.1016/S0042-6822(03)00484-7 14554082

[B55] Hoffman NG Schiffer CA Swanstrom R Covariation of amino acid positions in HIV-1 protease (vol 314, pg 536, 2003) Virology 2005 331 206 207 10.1016/j.virol.2004.10.029 14554082

[B56] Rhee SY Liu T Ravela J Gonzales MJ Shafer RW Distribution of human immunodeficiency virus type 1 protease and reverse transcriptase mutation patterns in 4,183 persons undergoing genotypic resistance testing Antimicrob Agents Chemother 2004 48 3122 3126 10.1128/AAC.48.8.3122-3126.2004 15273130 PMC478552

[B57] Chen L Lee C Distinguishing HIV-1 drug resistance, accessory, and viral fitness mutations using conditional selection pressure analysis of treated versus untreated patient samples Biology Direct 2006 1 14 10.1186/1745-6150-1-14 16737543 PMC1523337

[B58] Liu Y Eyal E Bahar I Analysis of correlated mutations in HIV-1 protease using spectral clustering Bioinformatics 2008 24 1243 1250 10.1093/bioinformatics/btn110 18375964 PMC2373918

[B59] Rhee SY Liu TF Holmes SP Shafer RW HIV-1 subtype B protease and reverse transcriptase amino acid covariation PLoS Computational Biology 2007 3 836 843 10.1371/journal.pcbi.0030087 PMC1866358 17500586

[B60] Wang Q Lee C Distinguishing Functional Amino Acid Covariation from Background Linkage Disequilibrium in HIV Protease and Reverse Transcriptase PLoS One 2007 2 e814 10.1371/journal.pone.0000814 17726544 PMC1950573

[B61] Haq O Levy RM Morozov AV Andrec M Pairwise and higher-order correlations among drug-resistance mutations in HIV-1 subtype B protease BMC Bioinformatics 2009 10 Suppl 8 S10 10.1186/1471-2105-10-S8-S10 19758465 PMC2745583

[B62] Reuman EC Rhee SY Holmes SP Shafer RW Constrained patterns of covariation and clustering of HIV-1 non-nucleoside reverse transcriptase inhibitor resistance mutations Journal of Antimicrobial Chemotherapy 2010 65 1477 1485 10.1093/jac/dkq140 20462946 PMC2882873

[B63] Zhang J Hou TJ Wang W Liu JS Detecting and understanding combinatorial mutation patterns responsible for HIV drug resistance Proceedings of the National Academy of Sciences of the United States of America 2010 107 1321 1326 10.1073/pnas.0907304107 20080674 PMC2824344

[B64] Beerenwinkel N Daumer M Oette M Korn K Hoffmann D Kaiser R Lengauer T Selbig J Walter H Geno2pheno: estimating phenotypic drug resistance from HIV-1 genotypes Nucleic Acids Research 2003 31 3850 3855 10.1093/nar/gkg575 12824435 PMC168981

[B65] Wang K Jenwitheesuk E Samudrala R Mittler JE Simple linear model provides highly accurate genotypic predictions of HIV-1 drug resistance Antiviral Therapy 2004 9 343 352 15259897

[B66] Jenwitheesuk E Wang K Mittler JE Samudrala R PIRSpred: a web server for reliable HIV-1 protein-inhibitor resistance/susceptibility prediction Trends in Microbiology 2005 13 150 151 10.1016/j.tim.2005.02.003 15817383

[B67] Rabinowitz M Myers L Banjevic M Chan A Sweetkind-Singer J Haberer J McCann K Wolkowicz R Accurate prediction of HIV-1 drug response from the reverse transcriptase and protease amino acid sequences using sparse models created by convex optimization Bioinformatics 2006 22 541 549 10.1093/bioinformatics/btk011 16368772

[B68] Saigo H Uno T Tsuda K Mining complex genotypic features for predicting HIV-1 drug resistance Bioinformatics 2007 23 2455 2462 10.1093/bioinformatics/btm353 17698858

[B69] Vermeiren H Van Craenenbroeck E Alen P Bacheler L Picchio G Lecocq P Prediction of HIV-1 drug susceptibility phenotype from the viral genotype using linear regression modeling Journal of Virological Methods 2007 145 47 55 10.1016/j.jviromet.2007.05.009 17574687

[B70] Beerenwinkel N Schmidt B Walter H Kaiser R Lengauer T Hoffmann D Korn K Selbig J Diversity and complexity of HIV-1 drug resistance: A bioinformatics approach to predicting phenotype from genotype Proceedings of the National Academy of Sciences of the United States of America 2002 99 8271 8276 10.1073/pnas.112177799 12060770 PMC123057

[B71] Sevin AD DeGruttola V Nijhuis M Schapiro JM Foulkes AS Para MF Boucher CAB Methods for investigation of the relationship between drug-susceptibility phenotype and human immunodeficiency virus type 1 genotype with applications to AIDS Clinical Trials Group 333 Journal of Infectious Diseases 2000 182 59 67 10.1086/315673 10882582

[B72] Srisawat A Kijsirikul B Combining classifiers for HIV-1 drug resistance prediction Protein and Peptide Letters 2008 15 435 442 10.2174/092986608784567537 18537731

[B73] Wang D Larder B Revell A Montaner J Harrigan R De Wolf F Lange J Wegner S Ruiz L Perez-Elias MJ Emery S Gatell J Monforte AD Torti C Zazzi M Lane C A comparison of three computational modelling methods for the prediction of virological response to combination HIV therapy Artificial Intelligence in Medicine 2009 47 63 74 10.1016/j.artmed.2009.05.002 19524413

[B74] Heider D Verheyen J Hoffmann D Machine learning on normalized protein sequences BMC Research Notes 2011 4 94 10.1186/1756-0500-4-94 21453485 PMC3079662

[B75] Rhee SY Taylor J Wadhera G Ben-Hur A Brutlag DL Shafer RW Genotypic predictors of human immunodeficiency virus type 1 drug resistance Proceedings of the National Academy of Sciences of the United States of America 2006 103 17355 17360 10.1073/pnas.0607274103 17065321 PMC1622926

[B76] Beerenwinkel N Lengauer T Daumer M Kaiser R Walter H Korn K Hoffmann D Selbig J Methods for optimizing antiviral combination therapies Bioinformatics 2003 19 I16 i25 10.1093/bioinformatics/btg1001 12855433

[B77] Altmann A Beerenwinkel N Sing T Savenkov I Daumer M Kaiser R Rhee SY Fessel WJ Shafer RW Lengauer T Improved prediction of response to antiretroviral combination therapy using the genetic barrier to drug resistance Antiviral Therapy 2007 12 169 178 17503659 10.1177/135965350701200202

[B78] Larder B Wang DC Revell A Montaner J Harrigan R De Wolf F Lange J Wegner S Ruiz L Perez-Elias MJ Emery S Gatell J Monforte AD Torti C Zazzi M Lane C The development of artificial neural networks to predict virological response to combination HIV therapy Antiviral Therapy 2007 12 15 24 17503743

[B79] Wittkop L Commenges D Pellegrin I Breilh D Neau D Lacoste D Pellegrin JL Chene G Dabis F Thiebaut R Alternative methods to analyse the impact of HIV mutations on virological response to antiviral therapy BMC Medical Research Methodology 2008 8 68 10.1186/1471-2288-8-68 18945369 PMC2605450

[B80] Altmann A Daumer M Beerenwinkel N Peres Y Schulter E Buch J Rhee SY Sonnerborg A Fessel WJ Shafer RW Zazzi M Kaiser R Lengauer T Predicting the Response to Combination Antiretroviral Therapy: Retrospective Validation of geno2pheno-THEO on a Large Clinical Database Journal of Infectious Diseases 2009 199 999 1006 10.1086/597305 19239365

[B81] Draghici S Potter RB Predicting HIV drug resistance with neural networks Bioinformatics 2003 19 98 107 10.1093/bioinformatics/19.1.98 12499299

[B82] Hou TJ Zhang W Wang J Wang W Predicting drug resistance of the HIV-1 protease using molecular interaction energy components Proteins-Structure Function and Bioinformatics 2009 74 837 846 10.1002/prot.22192 PMC3210569 18704937

[B83] Liu TF Shafer RW Web resources for HIV type 1 genotypic-resistance test interpretation Clinical Infectious Diseases 2006 42 1608 1618 10.1086/503914 16652319 PMC2547473

[B84] Rhee SY Fessel WJ Liu TF Marlowe NM Rowland CM Rode RA Vandamme AM Van Laethem K Brun-Vezinet F Calvez V Taylor J Hurley L Horberg M Shafer RW Predictive Value of HIV-1 Genotypic Resistance Test Interpretation Algorithms Journal of Infectious Diseases 2009 200 453 463 10.1086/600073 19552527 PMC4774893

[B85] Petropoulos CJ Parkin NT Limoli KL Lie YS Wrin T Huang W Tian H Smith D Winslow GA Capon DJ Whitcomb JM A novel phenotypic drug susceptibility assay for human immunodeficiency virus type 1 Antimicrob Agents Chemother 2000 44 920 928 10.1128/AAC.44.4.920-928.2000 10722492 PMC89793

[B86] Vinod HD Integer programming and theory of grouping Journal of the American Statistical Association 1969 64 506 519 10.2307/2283635

[B87] Rao MR Cluster analysis and mathematical programming Journal of the American Statistical Association 1971 66 622 626 10.2307/2283542

[B88] Bradley PS Fayyad UM Mangasarian OL Mathematical programming for data mining: Formulations and challenges Informs Journal on Computing 1999 11 217 238 10.1287/ijoc.11.3.217

[B89] Saglam B Salman FS Sayin S Turkay M A mixed-integer programming approach to the clustering problem with an application in customer segmentation European Journal of Operational Research 2006 173 866 879 10.1016/j.ejor.2005.04.048

[B90] Tseng GC Wong WH Tight clustering: A resampling-based approach for identifying stable and tight patterns in data Biometrics 2005 61 10 16 10.1111/j.0006-341X.2005.031032.x 15737073

[B91] Tibshirani R Walther G Hastie T Estimating the number of clusters in a data set via the gap statistic Journal of the Royal Statistical Society Series B-Statistical Methodology 2001 63 411 423 10.1111/1467-9868.00293

[B92] Hastie T Tibshirani R Friedman J The Elements of Statistical Learning 2009 2 New York, NY: Springer

[B93] King BM Tidor B MIST: Maximum Information Spanning Trees for dimension reduction of biological data sets Bioinformatics 2009 25 1165 1172 10.1093/bioinformatics/btp109 19261718 PMC2672626

[B94] Diaconis P Goel S Holmes S Horseshoes in multidimensional scaling and local kernel methods Annals of Applied Statistics 2008 2 777 807

[B95] HIV Drug Resistance Database http://hivdb.stanford.edu/cgi-bin/PIResiNote.cgi

[B96] Mammano F Trouplin V Zennou V Clavel F Retracing the evolutionary pathways of human immunodeficiency virus type 1 resistance to protease inhibitors: Virus fitness in the absence and in the presence of drug J Virol 2000 74 8524 8531 10.1128/JVI.74.18.8524-8531.2000 10954553 PMC116364

[B97] de Meyer S Vangeneugden T van Baelen B de Paepe E van Marck H Picchio G Lefebvre E de Bethune MP Resistance profile of darunavir: Combined 24-week results from the POWER trials Aids Res Hum Retrovir 2008 24 379 388 10.1089/aid.2007.0173 18327986

[B98] Nalam MNL Peeters A Jonckers THM Dierynck I Schiffer CA Crystal structure of lysine sulfonamide inhibitor reveals the displacement of the conserved flap water molecule in human immunodeficiency virus type 1 protease J Virol 2007 81 9512 9518 10.1128/JVI.00799-07 17596316 PMC1951406

[B99] Hertogs K de Bethune MP Miller V Ivens T Schel P Van Cauwenberge A Van den Eynde C Van Gerwen V Azijn H Van Houtte M Peeters F Staszewski S Conant M Bloor S Kemp S Larder B Pauwels R A rapid method for simultaneous detection of phenotypic resistance to inhibitors of protease and reverse transcriptase in recombinant human immunodeficiency virus type 1 isolates from patients treated with antiretroviral drugs Antimicrob Agents Chemother 1998 42 269 276 10.1093/jac/42.2.269 9527771 PMC105399

