# Supplementary material for: A multifaceted analysis of HIV-1 protease multidrug resistance phenotypes
Source: BMC Bioinformatics. 2011 Dec 15;12:477. doi: 10.1186/1471-2105-12-477 (PMC3305535; doi:10.1186/1471-2105-12-477)
Supplement: Additional file 1 — Contains the formal integer-programming formulations used within the work, five supplementary figures (Figures S1-S5) and one supplementary table (Table S1). This file also contains a link to a website containing the n = 398 data set used in this work. (http://www.wellesley.edu/Chemistry/Radhakrishnan/projects.html). [file 1471-2105-12-477-S1.PDF]

SUPPLEMENTARY MATERIAL: Doherty et. al., "A multifaceted analysis of HIV-1 protease multidrug resistance phenotypes"

Supplementary Methods:

Data set: The raw n=398 data set is available for download at http://www.wellesley.edu/Chemistry/Radhakrishnan/projects.html. We note that most sequences are not in GenBank because they were amino acid sequences obtained from papers, pharmaceutical companies, and other collaborators (see Rhee *et al., Antimicrob. Agents Chemother.,* 54:4253-4261,2010, as cited in our text).

Integer-programming formulations for phenotypic clustering and selection of representative sequences: We implemented a globally optimal, constrained k-medoids methods using an linear integer-programming approach. This implementation guarantees the optimality of the clustering obtained. Our data set was of a size such that obtaining a solution using an integer-programming formulation was fast (~15 seconds on a typical desktop workstation).

The integer programming formulation used for phenotypic clustering :
Parameters:
C: Maximum allowed Euclidean 2-norm distance between a sequence and its assigned medoid.
C_∞: Maximum allowed infinity-norm distance between a sequence and its assigned medoid.
$d(i_1, i_2)$: 2-norm distance between sequences i_1 and i_2
$d_\infty(i_1,i_2)$: infinity-norm distance between sequences i_1 and i_2
B: A large constant set greater than the maximum possible distance between any two sequences ($B=100$ in this work).

Binary Variables:
$y(i_1) = 1$ iff sequence i_1 is chosen as a medoid; 0 otherwise.
$z(i_1, i_2) = 1$ iff sequence i_2 is assigned to the cluster whose medoid is i_1, 0 otherwise.

Minimize $\sum_{i_1}\sum_{i_2} z(i_1,i_2)\times d(i_1,i_2)$ (minimize sum of all distances to medoids)

Such that:

$\sum_{i_1} y(i_i) = k$ (number of medoids selected = k, the number of clusters)

$\forall i_2, \sum_{i_1} z(i_i,i_2) = 1$ (each sequence assigned to exactly one cluster)

$\forall i_1,i_2, z(i_1,i_2) \le y(i_1)$ ($z(i_1, i_2) = 1$ iff sequence i_2 is assigned to medoid i_1)

$\forall i_1,i_2, y(i_1)+z(i_1,i_2)-2 \le \left(\frac{1}{B}\right)\times\left(C-d(i_1,i_2)\right)$ (i_2 is within distance C to its medoid)

$\forall i_1,i_2, y(i_1)+z(i_1,i_2)-2 \le \left(\frac{1}{B}\right)\times\left(C_\infty-d_\infty(i_1,i_2)\right)$ (i_2 is w/in infinity norm C_∞ to its medoid)

The integer-programming formulation for selecting representative sequences within a phenotypic cluster:

Definition:
Let M be set of sequences that contain mixtures at relevant positions (see Methods).

Parameters:
C: Maximum allowed genotypic distance between a sequence and its assigned medoid.
$d(i_1, i_2)$: Genotypic distance between sequences i_1 and i_2
B: A large constant set greater than the maximum possible distance between any two sequences ($B=100$ here).

Binary Variables:
$y(i_1) = 1$ iff sequence i_1 is chosen as a medoid; 0 otherwise.
$z(i_1, i_2) = 1$ iff sequence i_2 is assigned to the cluster whose medoid is i_1, 0 otherwise.

Minimize $\sum_{i_1}\sum_{i_2} z(i_1,i_2)\times d(i_1,i_2)$ (minimize sum of all distances to medoids)

Such that:

$\sum_{i_1} y(i_i) = k$ (number of medoids selected = k, the number of clusters)

$\forall i_2, \sum_{i_1} z(i_i,i_2) = 1$ (each sequence assigned to exactly one medoid)

$\forall i_1,i_2, z(i_1,i_2) \le y(i_1)$ ($z(i_1, i_2) = 1$ iff sequence i_2 is assigned to medoid i_1)

$\forall i_1,i_2, y(i_1)+z(i_1,i_2)-2 \le \left(\frac{1}{B}\right)\times\left(C-d(i_1,i_2)\right)$ (i_2 is within distance C to its medoid)

$\forall i_1 \in M, y(i_1) = 0$ (mixtures cannot be representative sequences)

Supplementary Figures:

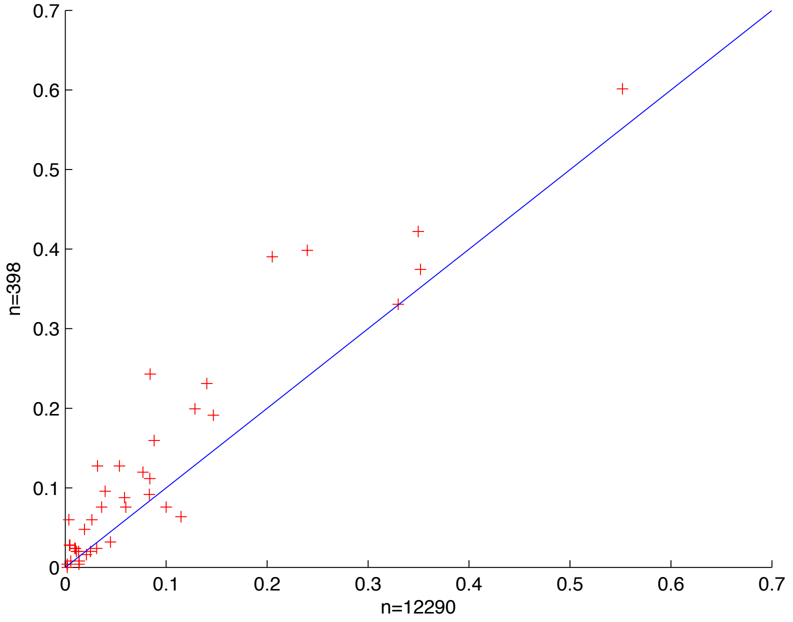

Figure S1: Mutation frequencies in the genotype/phenotype data set used in this work (n=398) for selected mutations vs. the frequencies within a larger genotype-only data set (n=12,290)[1]. Spearman's ρ=0.88 for this plot. The mutations considered were 10F, 24I, 30N, 32I, 33F, 43T, 46I, 46L, 47A, 47V, 48M, 48V, 50L, 50V, 53L, 54A, 54L, 54M, 54S, 54T, 54V, 71I, 71L, 73C, 73S, 73T, 74P, 76V, 82A, 82F, 82L, 82S, 82T, 83D, 84A, 84C, 84V, 88D, 88S, 90M. Wild type (WT) was defined as a sequence that did not contain any of the above mutations. If an isolate in the data set used for this work was a mixture that contained the mutation, it was counted as being present for that sequence.

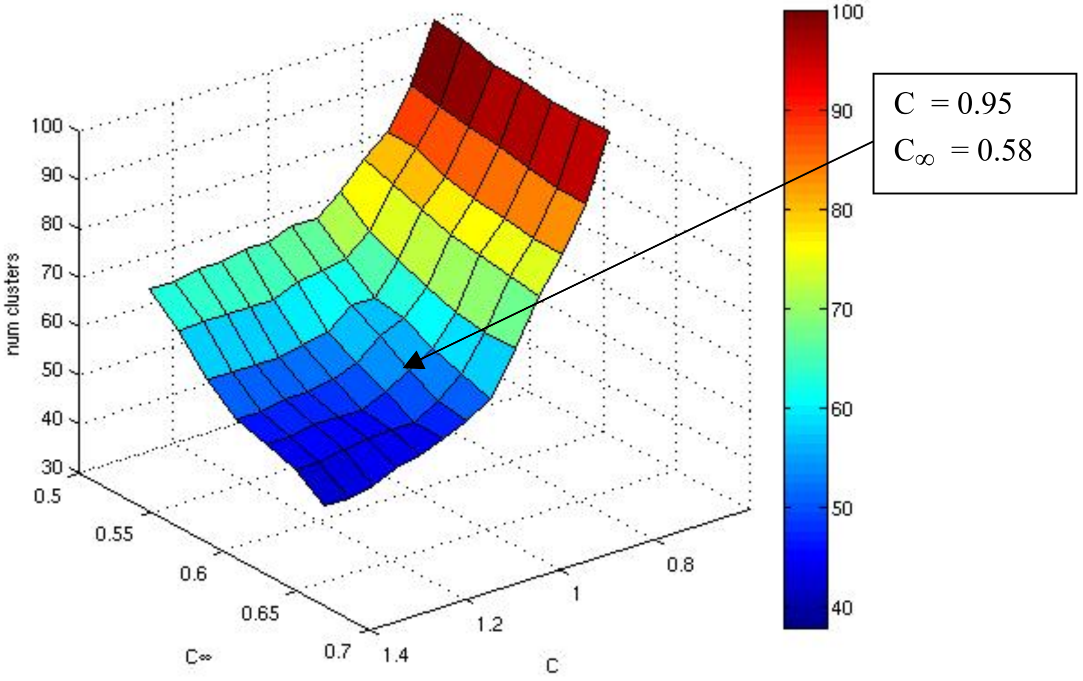

Figure S2: The minimum number of clusters needed given varying constraints for distance (C) and maximum dimensional distance (C_∞).

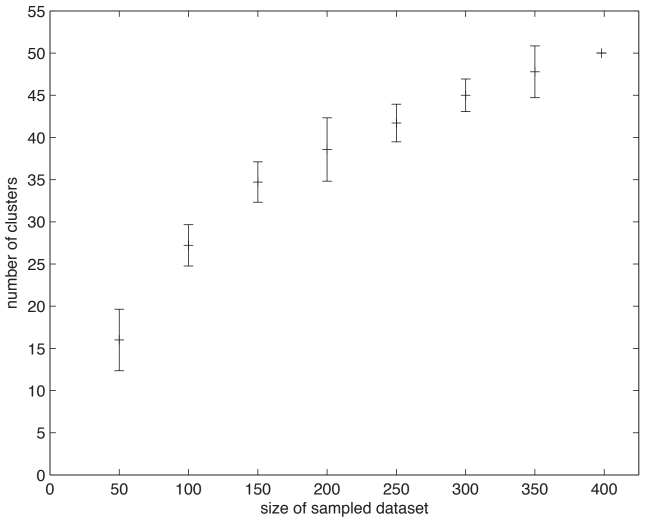

Figure S3: The minimum number of clusters needed as a function of data set size. Random subsets of the actual n=398 data set were generated. Fourteen trials were done at each point, with the (mean +/- one standard deviation) shown.

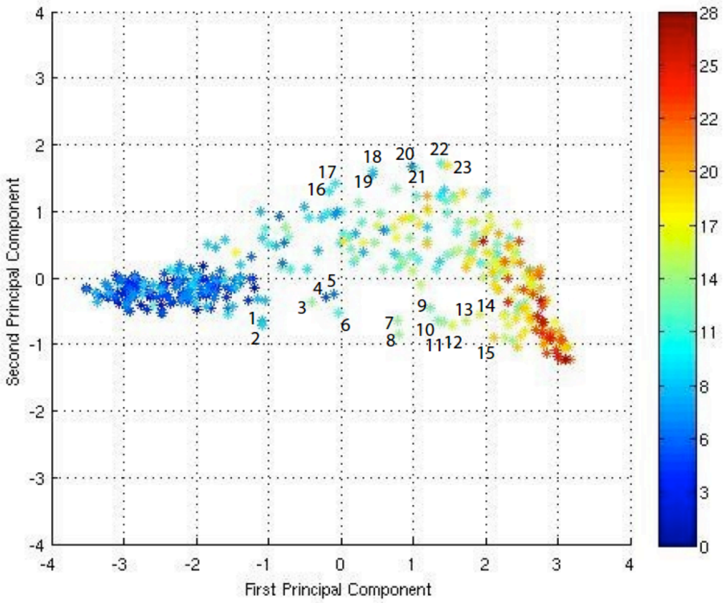

FIGURE S4: Projection of the phenotypic data onto its first and second principal components. See caption of Fig. 2. Here, a large number of "outliers" are numbered and their 9-digit phenotypic "code" and genotypes are shown below. The order of drugs used in the phenotypic code is RTV, NFV, ATV, APV, IDV,LPV, SQV, TPV, DRV. Outliers tend to fall into two groups: those that maintain "unique" susceptibility to SQV and have either V82L or I47A present, and those that show low levels of resistance to all or most drugs, including DRV. Points 16-23 form the "edge" of the path, and they show resistance to multiple drugs but no resistance or hypersusceptibility to both TPV and DRV.

1) 121111121: L10I , K14RK , G16EG , G17EG , R57K , L63T , A71T , I93L
2) 211201121: T4NT , I13V , L33F , M36I , R41K , R57K , E65D , H69K , L89M
3) 321323011: L10IL , T12SP , L19I , V32I , E35D , M36I , R41KR , M46LM , I47A , Q61HQ , L63P , I64V , A71VA , I93L
4) 422211121: L33F , K43T , L63C , H69Q , V77I
5) 421311111: L10I , I15V , I54L , I62V , L63P , I93L
6) 432111041: L33F , M36L , P39Q , R41K , K43R , I54V , I62V , L63P , A71V , V82L , I93L
7) 433322132: I13VI , V32IV , L33F , E35D , M36IM , R41K , I47VI , K55N , R57K , L63P , I64M , A71VA , L90M , I93LI
8) 443312041: L33F , M36L , P39Q , R41K , K43R , M46I , I47V , F53L , I54V , I62V , L63P , A71V , V82L , I93L
9) 444332131: L10I , I13VI , V32I , E35D , M36IM , N37S , K43T , M46I , I47V , L63P , V77IV , V82T , I85V
10) 442434022: L10I , I13V , V32I , L33F , M36I , R41K , I47A , L63P , K70E , A71V , V82A , L90M , I93L
11) 443434022: L10TI , I13V , V32I , L33F , M36I , R41KR , M46LIM , I47A , L63P , K70E , A71V , V82A , L90M , I93L
12) 443434032: L10V , I13VI , I15V , L19I , V32I , L33FL , M46I , I47A , K55NK , Q58EQ , L63P , K70EK , A71V , V77I , V82A , I93L
13) 444322232: L10I , V11I , I13V , A22V , V32I , R41K , M46I , K55R , I62V , L63P , I64V , H69Q , A71V , V82A , I84V
14) 443444022: L10I , G16A , L19Q , V32I , N37S , R41K , K45R , M46I , I47A , L63P , A71V , I72V , V82A , L90M , Q92K , I93L
15) 444444032: L10V , T12ATEK , K14RK , I15VI , Q18EQ , K20RK , V32I , L33FL , E34QE , E35DE , M36I , M46I , I47A , R57K , L63P , A71V , I72V , V82A , I93L
16) 442121200: L10I , I13V , K20T , D60E , L63P , C67D , A71V , V77I , L90M , I93L
17) 442121200: L23IL , V32IV , I50LI , I62V , L63P , A71VA , G73CS , V77I , L90M , I93L
18) 443131300: L10I , T12E , K20I , E35D , I62V , L63P , A71V , G73S , V77I , L90M
19) 443121300: K20V , F53L , L63P , A71V , G73S , V77I , I85VI , L90M
20) 443132400: L10I , L19IT , G48V , I54V , I64V , V82A , L90M
21) 444131400: L10I , I13V , K20I , L33V , M46L , L63P , A71T , I72V , T74K , I85V , N88D , L90M , I93L
22) 444143400: L10I , N37D , G48V , F53L , I54V , L63P , A71T , V77I , V82A , L90M , I93L
23) 444043400: L10I , I15V , L19I , K20M , L24I , E35D , M36I , G48Q , F53L , I54V , L63P , K70E , A71V , I72M , G73S , V75IV , V82A , I93MI

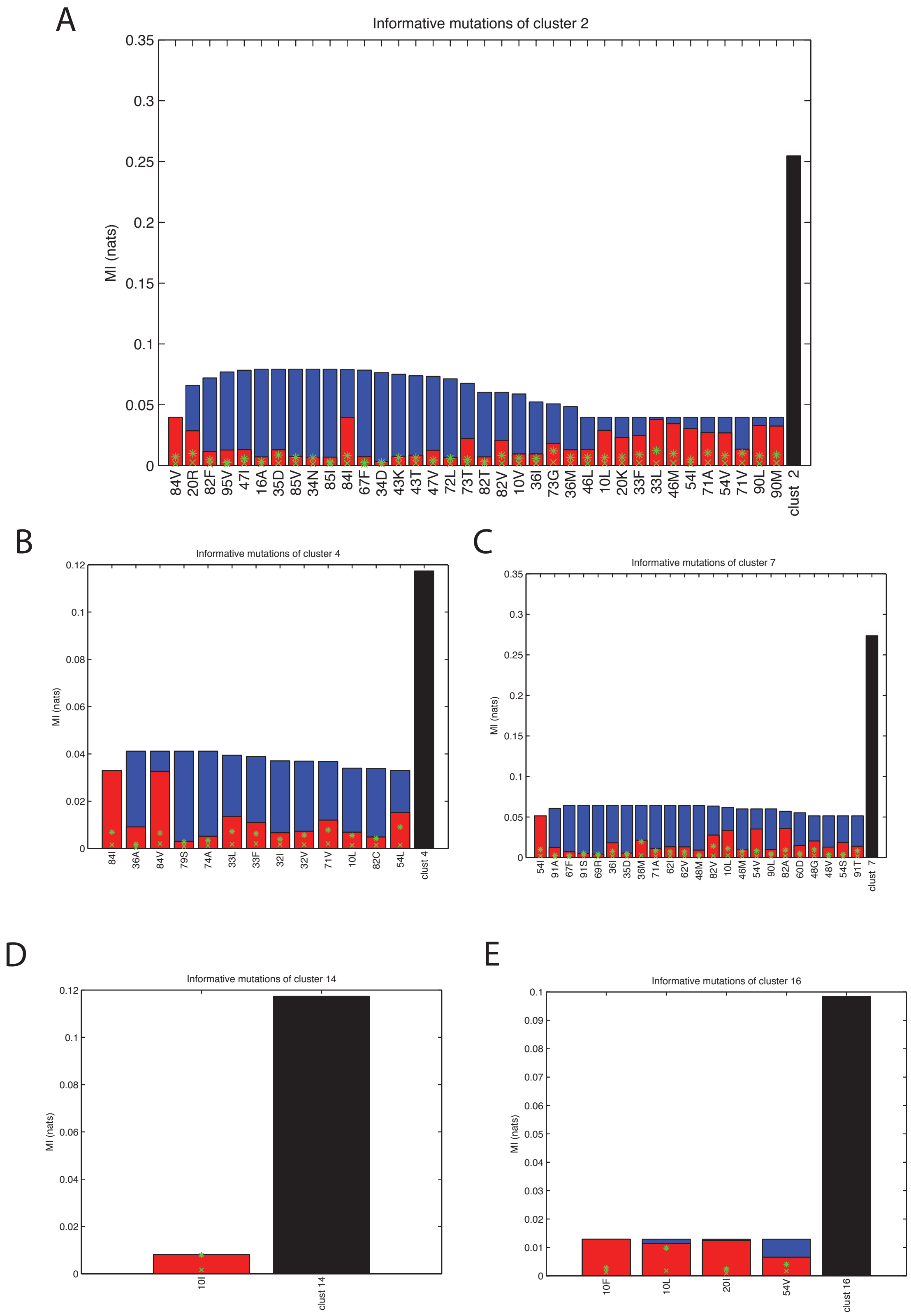

A
Informative mutations of cluster 2
MI (nats)
0 0.05 0.1 0.15 0.2 0.25 0.3 0.35
84V 20R 82F 95V 47I 16A 35D 85V 34N 85I 84I 67F 34D 43K 43T 47V 72L 73T 82T 82V 10V 36I 73G 36M 46L 10L 20K 33F 33L 46M 54I 71A 54V 71V 90L 90M clust 2
B
Informative mutations of cluster 4
MI (nats)
0 0.02 0.04 0.06 0.08 0.1 0.12
84I 36A 84V 79S 74A 33L 33F 32I 32V 71V 10L 82C 54L clust 4
C
Informative mutations of cluster 7
MI (nats)
0 0.05 0.1 0.15 0.2 0.25 0.3 0.35
54I 91A 67F 91S 69R 36I 35D 36M 71A 62I 62V 48M 82V 10L 46M 54V 90L 82A 60D 48G 48V 54S 91T clust 7
D
Informative mutations of cluster 14
MI (nats)
0 0.02 0.04 0.06 0.08 0.1 0.12
10I clust 14
E
Informative mutations of cluster 16
MI (nats)
0 0.01 0.02 0.03 0.04 0.05 0.06 0.07 0.08 0.09 0.1
10F 10L 20I 54V clust 16

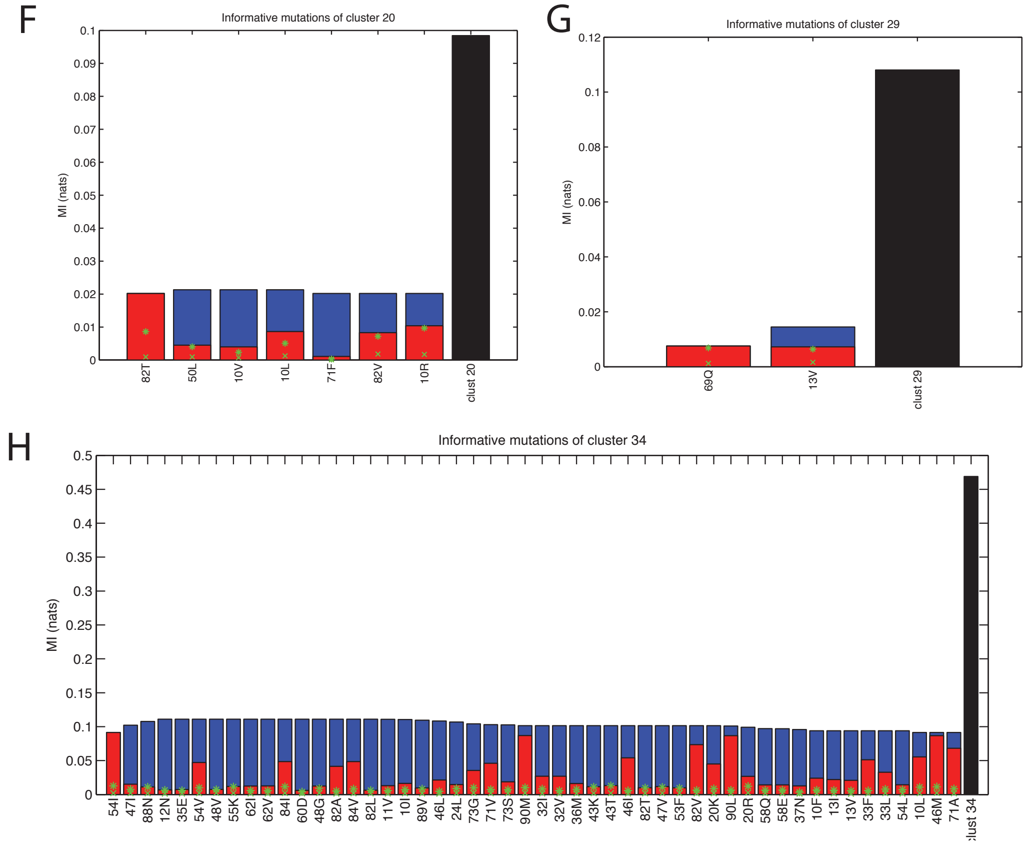

Figure S5: Stepwise selection of informative amino acid identities for selection into specific phenotypic clusters. All clusters with 8+ members that have at least one mutation with statistically significant MI with the cluster assignment are shown, excluding those shown in the main article (clusters 1 and 36). An explanation of these plots is in the caption of Figure 4 of the main article and in the main text. Plots are shown for Clusters (a) 2 , (b) 4, (c) 7, (d) 14, (e) 16, (f) 20, (g) 29, (h) 34. The phenotype of each cluster is indicated in the main article.

Table S1: complete mutation listing for all representative sequences (assuming subtype B consensus as WT)

Num	Size	Representative Sequence(s)
1	15	L10I , V11I , T12K , I13V , K20V , V32I , L33F , E35G , M36I , N37D , M46I , I47V , I54M , R57K , Q58E , L63P , I64V , I66V , A71V , G73S, I84V, L89V, L90M
2	28	L10F , G16A , L19I , K20R , L33F , E35D , M36L , N37D , K43T , M46L , I54V , K55R , R57K , D60E , I62V , L63P , A71V , V82A , I84V , L90M , I93L
3	4	L10I , K20R , L24I , E35D , M36I , N37E , M46I , I54V , Q61N , I62V , L63P , I64V , A71V , I72V , T74S , L76V , V82T , I93L
4	10	V32I , L33F , E34Q , M36A , P39S , M46I , F53L , I54L , K55RK , I62V , L63P , A71V , I84V , L90M
5	6	L10F , I13V , K20R , D30N , V32I , L33F , M36I , N37D , M46I , I54L , L63P , I64L , I66V , A71T , I72T , I84V , N88D , L89M , L90M , I93L , C95F
		L10I, K20R, L33F, E34T, E35D, M36I, N37D, K43T, M46I, G48V, I50V, I54S, R57K, I62V, L63P, A71V, I72V, V77I, V82A, I85V, I93L
6	6	L10I , V11I , I13V , A22V , V32I , R41K , M46I , K55R , I62V , L63P , I64V , H69Q , A71V , V82A , I84V
7	31	L10I , K20R , E35D , M36I , N37D , R41K , M46I , I54V , I62V , L63P , V82A , L90M , I93L , C95F
8	3	L10F , V11I , I15V , K20V , E34QR , E35D , M36I , N37S , R41K , G48I , I54L , K55R , Q58E , D60NS , I62V , L63P , A71I , I72MI , G73S , L76V , I84V , L90M , I93L
9	7	: L10I , I13V , I15V , L19V , L24I , V32I , L33F , K43E , M46L , I54L , D60E , L63P , A71V , I72V , V82A , I84V
		L10F , I13V , K20T , L24I , D30N , L33F , E35D , M36I , N37S , K43T , F53L , K55N , L63P , V77IV , N88D , L89M
10	4	L10I, K20R, E35D, M36I, K43T, I47V, G48M, F53L, I54V, I62V, L63P, I66F, A71V, V82T, I84V, Q92R
11	2	L10I , G16A , L19Q , V32I , N37S , R41K , K45R , M46I , I47A , L63P , A71V , I72V , V82A , L90M , Q92K , I93L
12	6	L10I , G16A , L33F , K45R , I54L , K55R , I62V , L63P , C67YC , I72L , G73C , V77I , I84V , L90M , I93L
13	5	L10I , V11I , T12PT , I13V , K20RK , E35D , M36I , R41K , M46L , I62VI , L63P , A71V , I84V , L90M , Q92K , I93L
14	10	L10I , I15V , L19I , K20R , E35D , M36I , N37E , R41K , I54V , Q58E , D60N , I62V , L63P , K70R , A71V , G73S , V82A , L90M , I93L
15	3	L10I , I13V , V32I , L33F , M36I , R41K , I47A , L63P , K70E , A71V , V82A , L90M , I93L
16	8	L10F , T12PT , I13V , K14RK , K20I , E35D , M36I , N37D , M46I , I54V , L63P , A71V , I72V , V82A , L90M , I93L
17	3	L10I , K20R , L24I , L33F , E35D , M36I , N37D , M46L , I54V , L63P , A71T , V82A , T91S
18	4	L10I , L19IT , G48V , I54V , I64V , V82A , L90M
19	4	L10I , I13VI , V32I , E35D , M36IM , N37S , K43T , M46I , I47V , L63P , V77IV , V82T , I85V
20	8	L10I , K20R , E35D , M36I , N37D , I54V , R57K , D60E , L63P , A71V , I72T , V77IV , V82A , I93L
21	5	L10I , I13V , N37DN , R41K , M46I , I47V , L63P , A71T , I72V , V77I , N88G , L90M
22	2	L10I , T12KT , L33F , K43T , M46I , I50L , I54V , K55R , I62V , L63P , A71V , I72L , G73T , V77I , V82A , L90M , I93L , C95F
23	2	T4P , L10F , Q18HQ , L19I , L33F , E34Q , N37D , M46I , I54L , L63P , A71I , I72L , V77I , N88G , L90M , I93L
24	5	L10I , I13V , K20L , M46I , Q58E , I62V , L63P , A71T , T74S , V77I , N88S , L90M , Q92K
25	3	L10I , I13V , K14R , G16E , K20I , E35D , M36I , N37D , R41N , I54V , L63H , H69K , K70R , T74S , L76V , V82A , L89I , T91S
26	13	R57K , D60E , L63P , I64V , G73C , V77I , L90M
27	5	L10I , M46L , I62V , L63P , I85V , L90M , I93L
28	4	L10I , I13V , K20T , D60E , L63P , C67D , A71V , V77I , L90M , I93L
29	9	I13VI , K14RK , N37CS , R41KR , I64V , V77I
30	2	L10H , I13VI , Q18H , L19IM , K20T , R41K , M46I , D60E , L63P , A71I , V77I , I93L , C95YC
31	4	I15V , G16E , D60E , Q61EQ , I62V , L63KQ , I72EV , V77I
32	5	L10I , L24I , R41K , M46I , I54V , I64V , V77I , V82A
33	4	I13VI , G16EG , L19V , K20I , L63P , K70R , A71T , I72VI
34	71	K14R , I15V , G16E , K20TK , M36I , P39S , D60E , I62V , L63P , I72V
35	6	I15V , L19T , K20R , M36I , R41K , I62V , K70R , V82L
36	77	R57K , V77I
37	1	L10F , G16AG , K20RK , V32I , L33F , M36IML , N37DN , M46I , I47V , I54M , Q58E , L63P , A71V , V82A , I93L
38	1	L10I , E35D , M36I , G48V , I50V , I54V , I62V , L63A , A71V , V82T
39	1	L10I , N37CNSY , M46I , I54LIM , I62V , L63P , I64V , A71V , I72TI , L76V , V77I , I84V , I85V , G86RG , I93L
40	1	L10I , D30N , L33F , M46L , F53L , I54L , Q58E , L63P , A71T , G73S , V77I , N88D
41	1	L33F , M36L , P39Q , R41K , K43R , M46I , I47V , F53L , I54V , I62V , L63P , A71V , V82L , I93L
42	1	L10I , I15V , L19I , K20R , M36I , R41K , M46L , I54V , L63P , A71V , L76V , V82A , L90M , I93L
43	1	L33F , M36L , P39Q , R41K , K43R , I54V , I62V , L63P , A71V , V82L , I93L
44	1	L10IL , T12SP , L19I , V32I , E35D , M36I , R41KR , M46LM , I47A , Q61HQ , L63P , I64V , A71VA , I93L
45	1	L10I , I15V , I54L , I62V , L63P , I93L
46	1	L10I , T12KT , K14R , L23I , L33VL , E35D , M36V , N37H , R41K, M46I , I50L , R57K , L63P , I64V , K70R , V82A , I85V
47	1	L10V , I13V , K20T , L33F , M46I , Q58E , I62V , L63P , V77I , N88G
48	1	D30N , L33F , I62V , L63P , I64L , A71VA , V77I , N88D , I93L
49	1	I13V , D30N , K43T , M46I , D60E , Q61E , L63P , V77I , I93L
50	1	K45R , I50L , I62V , L63P , A71IT , I72V , G73SG , V77I , V82A , I93L

Supplementary References:

1. Rhee SY, Gonzales MJ, Kantor R, Betts BJ, Ravela J, Shafer RW. Human immunodeficiency virus reverse transcriptase and protease sequence database. Nucleic Acids Research 2003;31:298-303.
